# Multiclass classification of thalassemia types using complete blood count and HPLC data with machine learning

**DOI:** 10.1038/s41598-025-06594-6

**Published:** 2025-07-21

**Authors:** Muhammad Umar Nasir, Muhammad Zubair, Muhammad Tahir Naseem, Tariq Shahzad, Ahmed Saeed, Khan Muhammad Adnan, Amir H. Gandomi

**Affiliations:** 1 Faculty of Computing, Riphah International University, Islamabad, Pakistan; 2 School of Computing, IVY CMS, Lahore, Pakistan; 3 School of Computing, Arden University, Conventry, UK; 4 Department of Electronic Engineering, Yeungnam University, Gyeongsan, 38541 Republic of Korea; 5 Department of Computer Engineering, COMSATS University Islamabad, Sahiwal Campus, Sahiwal, 57000 Pakistan; 6 Division of Computing Science and Mathematics, University of Stirling, Stirling, FK9 4LA Scotland, UK; 7 Department of Software, Faculty of AI and Software, Gachon University, Seongnam-si, 13120 Republic of Korea; 8 Faculty of Engineering and IT, University of Technology Sydney, Sydney, NSW 2007 Australia; 9 University Research and Innovation Centre (EKIK), Obuda University, Budapest, 1034 Hungary; 10 Department of Computer Science, Khazar University, Baku, Azerbaijan

**Keywords:** Alpha thalassemia, Beta thalassemia, Alpha major, Alpha minor, Beta major, Beta minor, XGBoost, SVM, KNN, Complete blood count (CBC), High-performance liquid chromatography (HPLC), Medical research, Computational science, Computer science, Scientific data

## Abstract

Mild to severe anemia is caused by thalassemia, a common genetic disorder affecting over 100 countries worldwide, that results from the abnormality of one or several of the four globin genes. This leads to chronic hemolytic anemia and disrupted synthesis of hemoglobin chains, iron overload, and poor erythropoiesis. Although the diagnosis of thalassemia has improved globally along with the treatment and transfusion support, it is still a major problem in diagnosing in high-prevalence areas like Pakistan. This work aims to assess the performance of numerous combinations of machine learning methods to detect alpha and beta-thalassemia in their minor and major types. These results are obtained from CBC and HPLC analysis. The analyzed models are K-nearest Neighbor (KNN), Support Vector Machine (SVM), and Extreme Gradient Boosting (XGBoost). The study aims to examine the effectiveness of the developed models in discriminating thalassemia variants, especially in the light of Pakistani patients’ data. The study found that XGBoost achieved the highest performance on both the CBC and HPLC datasets, with training accuracies of roughly 99.5% for CBC and 99.3% for HPLC. The test accuracy across both datasets was consistently high and thus the best model for detecting thalassemia in this research study. The imported SVM model, slightly less accurate than XGBoost, still has strong performance, particularly on the HPLC data where the cumulative testing accuracy of the model stood at 99.4%. As can be seen from the results, XGBoost specifically shows a very high accuracy of above 99% in the detection of thalassemia types using CBC and HPLC data for Pakistani patients. To the author’s knowledge, this research is the first to predict alpha and beta-thalassemia in its major and minor forms using these diagnostic reports. These models indicate that they can offer significant support in detecting thalassemia in resource-constrained settings such as Pakistan. If deep learning is incorporated, even greater accuracy could be achieved.

## Introduction

**Fig. 1:**
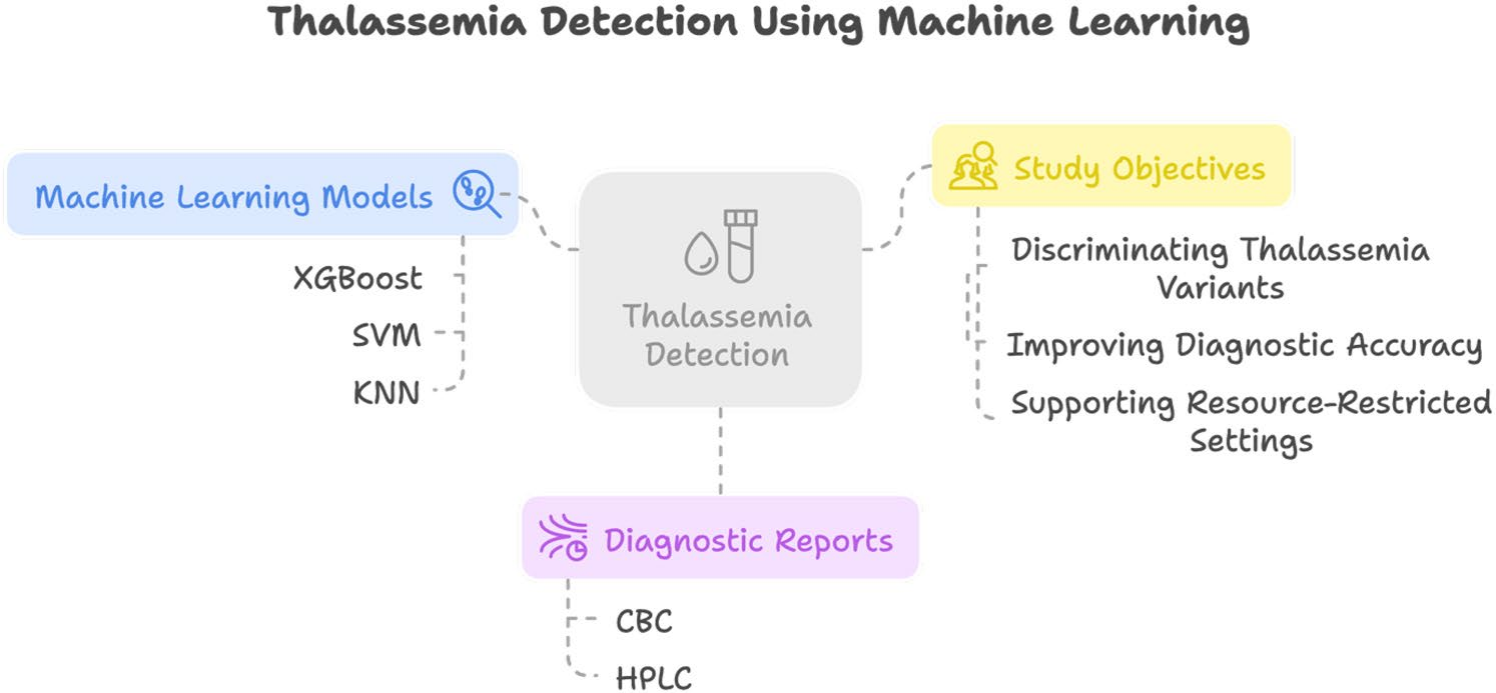
Graphical representation of abstract.

The term thalassemia is derived from the combination of two Greek words, Thalassa meaning Ocean and Haima meaning Blood^1^. Thalassemia is a genetic disorder that affects the blood by reducing hemoglobin production, a critical protein for transporting oxygen from the lungs to the body and carbon dioxide back to the lungs^2^. Figure 1 presents a graphical summary of the abstract, it shows study models, diagnostic reports and study outcomes.

Thalassemia is the most common genetic disorder, particularly prevalent in the Mediterranean region. Thalassemia is becoming increasingly prevalent in many regions across the globe, making it a significant burden on public health systems where it exists and a major cause of disability and mortality. This means that there is a need for HCPs to make informed decisions for early diagnosis to reduce mortality rates of the condition. Common in genetic disorders such as this, it is important to distinguish between healthy persons and persons whose genes contain the thalassemia gene^3^.

Thalassemia is categorized into two types based on the two polypeptide chains in hemoglobin: α and β thalassemia are two types of thalassemia known to humans. Alpha-thalassemia arises from mutations in the alpha-globin gene, while beta-thalassemia involves the beta-peptide gene^4^. Both types result in insufficient or abnormal hemoglobin production, impairing red blood cell function^5^.

Approximately 80,000–90,000 individuals in Pakistan are diagnosed with thalassemia and receive treatment through public and private health units^6^. Identifying asymptomatic thalassemia carriers, particularly those with the beta-thalassemia trait, is critical. When both parents are asymptomatic, the likelihood of their child being born with a severe form of thalassemia that requires a blood transfusion is one in four^7^. To diagnose thalassemia, one needs to take a life history of the patient, perform a clinical examination, blood samples for microscopy, and examination of different hemoglobin variants through hemoglobin electrophoresis. In this test, molecules are separated by charge in an electric field using a buffer, within which ions are between two electrodes. In healthy adults, the percentage prevalence of HbA is higher between 96 and 98% compared to HbA2 which is between 2 and 3.5%.

Thalassemia major is the most severe type of the disorder as it is combined with anemia and osteopenia^8^. Patients require regular transfusions to survive of blood products into the patient’s body. Nonetheless, chronic blood transfusions may cause sclerotized effects resulting from the accumulation of iron in the body to affect the heart, liver, and the endocrine system. This results in iron overload and since the body cannot tolerate excessive accumulation of iron, chelation therapy is used to help eliminate the excess iron^9–11^.

Numerous complications accompany thalassemia. Some of the problems include low sexual drive, low sperm density, reduced sperm quality, and among others^12^. Several researchers have found that blood transfusion therapy results in a rise in luteinizing hormone and follicle-stimulating hormone increasing sperm count and motility. Transfusions also help lower the risk of osteoporosis because blood transfusion increases secretion of insulin-like growth factor-1 (IGF-1) and IGF-binding protein-3^13,14^. IGF-1, a hormone involved in cell growth and protein metabolism, directly mediates growth hormone responses in tissues^15^. Furthermore, the literature reveals that a year of denosumab therapy enhances BMD in the L2–L4 and FN in BTM and lowers ICTP levels^16^.

The thyroid gland is also affected in thalassemia, with primary hypothyroidism as the most common complication due to glandular abnormalities. Secondary hypothyroidism from pituitary dysfunction is less common than primary hypothyroidism. In clinical practice, diagnosis is commonly made from biochemical tests with low levels of thyroid hormones and low or absent TSH^17^. Insulin resistance is another comorbidity, diagnosed most accurately using continuous glucose monitoring systems (CGMS) as the most accurate diagnostic method for insulin resistance and hyperglycemia in such patients^18^. In the course of the disease, thalassemia can also cause chronic kidney disease CKD as a result of the reduced kidney function seen in hyperfiltration, albuminuria, and renal damage^19,20^. Recent studies have identified the rapid progression of renal dysfunction in β-thalassemia major patients in which renal stress test (RST) has been proven to be a marker of renal dysfunction and iron overload along with acute kidney injury (AKI)^21^. Based on many indices of complete blood count including hemoglobin concentration (Hb), mean corpuscular hemoglobin (MCH), mean corpuscular hemoglobin concentration (MCHC) mean corpuscular volume (MCV), and red cell distribution width (RDW), thalassemia can be differentiated from other diseases.

The most common mutations involving the α-globin genes are deletions with the -α3.7 kb and  − α4.2 kb deletions the most prevalent^22^. The clinical and laboratory findings are similar in non-deletional mutations in the α genes. While in the East Asian countries, the α carrier genotype frequently presents the cis form (–/αα), the transform (− α/− α) is more observed in Western countries. This results in high incidences of hydrops fetalis and Hb H disease in the Asian population which is why screening for α-thalassemia carrier status is routine in Asian countries. Nonetheless, Hb H disease and hydrops fetalis also occur in Western populations with two or more α-globin gene deletions in the alpha gene (− α/-α)^22^. Antisense deletional defects are less severe than non-deletional defects, as the α2 gene produces more α-globin than the α1 gene^23^.

Some α-globin gene mutations have a synergistic effect with certain β-globin mutations and may either increase or decrease the severity of α-thalassemia. For instance, additional copies of α-thalassemia genes might worsen β-thalassemia to the extent of NTDT or even TDT^24^.

Machine learning has transformed data management and analysis across research fields. This has made it particularly beneficial in healthcare, where it can help improve diagnostic accuracy, enhance patient outcomes, and lower costs^25^. In the case of thalassemia, machine learning can offer more precise identification of the condition and better overall management^26^. Machine learning algorithms have successfully addressed biomedical challenges, with models developed for conditions like brain tumors^27^, kidney diseases^28^, lung disorders^29^, and iron deficiency anemia^30–32^. Techniques such as support vector machines^33^, K-nearest neighbors^34^, fuzzy logic^35–37^, deep extreme machine learning^38^, and deep neural networks^39^ have been applied.

While machine learning algorithms are now much better at diagnosing diseases, earlier versions faced challenges in accuracy. This was often due to their reliance on preprocessing methods, data balancing, and the use of supervised and semi-supervised learning techniques. Improving disease detection requires integrating data from diverse patient cases. The proposed model will focus on a feature-based dataset derived from thalassemia-related CBC and HPLC reports to achieve more accurate results.

The aim of this study was to evaluate the effectiveness of this screening approach and propose more effective and economical screening strategies. Additionally, we investigated whether the formulas outlined in existing literature are effective in identifying α thalassemia and beta thalassemia carriers using multiclass detection.

It highlights the need to accurately identify α and β-thalassemia carriers for timely intervention and genetic counselling. The paper discusses the limitations of current prediction models and the need for more effective methods. The objectives of the study are clearly outlined as follows:

- To develop machine learning-based techniques to detect alpha thalassemia and beta thalassemia both major and minor carriers using multiclass detection scenarios for both CBC and HPLC reports.
- To evaluate the performance of the proposed model using primary performance metrics and compare it with existing approaches.

## Literature review

Umar et al. ^26^ analysed patterns within both private and public thalassemia-related CBC datasets using machine learning (XGBoost) and deep learning (CNN) models to evaluate their classification performance. The study found that XGBoost achieved a highest accuracy of 99.34% on the private dataset for alpha thalassemia, while CNN attained 98.10% accuracy for beta thalassemia on the same dataset. However, their proposed models were not capable of distinguishing between the subtypes of alpha and beta thalassemia. Donghua et al.^40^ developed a deep neural network (DNN) model for detecting thalassemia, achieving an impressive 96% accuracy. The model was trained using feature-based data from 8693 patient records collected between 2014 and 2021. Despite its success, the model has limitations, including the small dataset size and the risk of overfitting. Additionally, the model lacked data on alpha-thalassemia and beta-thalassemia subtypes, limiting its ability to perform multiclass detection.

Shoaib et al.^41^ applied a Federated Learning (FL) model to detect beta-thalassemia, which achieved an accuracy of 92.38%. The model was trained on data from 5,066 patients. However, the study was limited by a small dataset and the absence of data on alpha-thalassemia and beta-thalassemia subtypes, restricting multiclass detection. Rustam et al.^42^ proposed a Convolutional Neural Network (CNN) model designed to detect beta-thalassemia carriers. The model, employing Principal Component Analysis (PCA) for feature selection, achieved 96% accuracy using data from 5066 self-reported patients. Despite strong performance, the study was limited by potential bias in self-reported data and the absence of alpha-thalassemia and beta-thalassemia subtype data, hindering multiclass detection.

Ucucu et al.^43^ Developed a model using K-nearest neighbors (KNN), Naïve Bayes, Decision Tree (DT), and the Boruta algorithm for feature selection to classify hemoglobin variants such as HbS and HbD. The model achieved an impressive 99% accuracy using data from 238 patients (90 women and 148 men) collected between 2015 and 2021. Despite its effectiveness, the model’s generalizability is limited by the small sample size (238 patients) and lack of data on alpha- and beta-thalassemia subtypes.

Feng et al.^44^ employed a Random Forest (RF) model to detect alpha-thalassemia, achieving an accuracy of 91.5%. The model was trained on data from 1213 patients, including 495 pregnant women, collected between 2018 and 2020. The study demonstrated the model’s capability in diagnosing alpha-thalassemia, with limited evaluation of beta-thalassemia subtypes. However, the regional focus of the dataset and the missing data on thalassemia subtypes with multiclass detection limit the broader applicability of the results.

ER Susanto et al.^45^ developed a Fuzzy Model for detecting thalassemia, although the study did not report specific accuracy figures. The model utilized feature-based data collected from patients. However, the study lacks detailed performance metrics and faces limitations in terms of generalizability. Additionally, it did not include data on alpha-thalassemia or beta-thalassemia (both minor and major subtypes).

Rena et al.^46^ created a machine-learning model that achieved an accuracy of 86.6%. The model was trained on feature-based data from 1076 samples to detect beta-thalassemia. Some limitations of the study include data incompleteness and the absence of information on alpha thalassemia and various beta thalassemia subtypes, especially multiclass detection.

Salman et al.^47^ used the MobilenetV2 model for image-based detection of alpha thalassemia, reaching an accuracy of 95.72%. The dataset consisted of 524 images collected over 2 years. This research demonstrated the potential of using image-based methods for thalassemia detection, especially in settings where medical images can be collected independently. However, the study’s reliance on image data could be a limitation, as such images might not be readily available in resource-limited environments.

Sadiq et al.^48^ developed an ensemble learning model for detecting beta-thalassemia, achieving a solid accuracy of 93%. The model was trained on data from 5066 self-reported cases. The study demonstrated the model’s effectiveness in detecting both alpha and beta thalassemia subtypes and its potential for clinical use. However, the generalizability of the results could be limited due to a smaller sample size and missing data on various thalassemia subtypes.

Fu et al.^49^ employed a support vector machine (SVM) model to detect thalassemia, achieving an area under the curve (AUC) of 0.76, indicating moderate diagnostic performance. The model was tested with a dataset of 350 patients collected between 2018 and 2020. While the study highlights the value of feature-based approaches in thalassemia detection, the relatively small dataset and the absence of data on alpha and beta thalassemia subtypes limit the model’s broader applicability.

Laengsri et al.^50^ implemented RF, KNN, and Artificial Neural Network (ANN) models to detect thalassemia, achieving an accuracy of 95.5% with a dataset of 186 patients collected between 2014 and 2016. The study emphasized the strength of feature-based approaches in detecting thalassemia variants. However, the relatively small sample size and missing data on alpha thalassemia, and beta thalassemia subtypes could limit the generalizability of the findings.

Monalisha et al.^51^ developed a KNN model for detecting hemoglobin variants in both alpha-thalassemia and beta-thalassemia cases, achieving a precision of 93.89%. The model was trained using feature-based data from 1500 samples. However, there were some limitations, including the relatively small sample size and potential biases arising from the self-collected nature of the data. Farhadi et al.^52^ explored the use of RF and DT models for thalassemia detection with data from 3489 cases collected in 2018. Their RF model achieved a sensitivity of 0.21 and a specificity of 0.77. Although the study aimed to detect thalassemia, the low sensitivity of the model limits its potential clinical application.

Jahangiri et al.^53^ developed a DT model for detecting beta-thalassemia, achieving an impressive AUC of 0.99. The model used self-collected feature-based data from 144 patients. However, there were some limitations, including missing data on alpha-thalassemia and various subtypes of beta-thalassemia. Kandhro et al.^54^ applied both DT and RF models, achieving a specificity of 90%. These models, based on self-collected feature data, were designed to detect both alpha-thalassemia and beta-thalassemia. The study faced limitations, including data incompleteness and a lack of information on various thalassemia subtypes and multiclass detection.

Risoluti et al.^55^ used a Partial Least Squares (PLS) model with a sensitivity of 89.9% to detect beta-thalassemia, using self-collected image data from 63 patients. The model showed promise, but there were limitations, such as missing data on alpha-thalassemia, and beta-thalassemia subtypes.

Matos et al.^56^ applied the Fisher Discriminant Index to detect both alpha-thalassemia and beta-thalassemia, achieving an accuracy of 99.3%. The model was trained on data from 185 patients. Despite the high accuracy, the study had limitations, such as missing information on thalassemia subtypes.

Huang et al.^57^ developed a model using 10 formulas to detect both alpha and beta-thalassemia, achieving a sensitivity of 89.62%. The model was applied to data from 877 patients. Limitations include missing details on thalassemia subtypes and multiclass detection.

Masala et al.^58^ created a model using KNN and PNN, which achieved a specificity of 91% in detecting alpha-thalassemia using self-collected data from 304 patients. The study faced limitations, including data incompleteness and missing information about thalassemia subtypes. Barnhart et al.^59^ employed an ANN model for detecting both alpha and beta-thalassemia, achieving a sensitivity of 0.897. The model was trained on feature-based data from 526 patients. However, the study had limitations, including missing data on thalassemia subtypes.

Janel et al.^60^ used 11 formulas to detect beta-thalassemia, achieving an accuracy of 93% with data from 129 patients. The study had limitations, such as missing information on alpha-thalassemia and beta-thalassemia subtypes, as well as multiclass detection.

Shen et al.^61^ applied 12 formulas for detecting beta-thalassemia, achieving an AUC of 0.947. The model was tested with data from 300 cases. However, limitations include missing data on thalassemia subtypes. Urrechaga et al.^62^ utilized a Multidimensional Analysis (MDA) model for detecting both alpha and beta-thalassemia, achieving an accuracy of 87.9% (with separate accuracy rates of 83.3% for beta-thalassemia and 72.1% for alpha-thalassemia). The model was based on feature-based data from 250 patients. Despite its usefulness, the study was limited by accuracy issues and missing data on thalassemia subtypes.

George et al.^63^ applied six formulas to detect beta-thalassemia, achieving a sensitivity of 75.06% with feature-based data from 373 patients. Limitations include missing data on thalassemia subtypes. Amendolia et al.^64^ developed a model using SVM, KNN, and MLP with a specificity of 95%. The model was trained on self-collected data from 304 records to detect thalassemia patients. However, multiple research studies demonstrated high accuracy yet they failed to detect multiple classes and included incomplete information about alpha thalassemia subtypes. The previous studies encountered various limitations because they did not provide comprehensive information about thalassemia subtypes. Table 1 depicts the limitations of previous studies.

**Table 1:** Limitations and results of previous studies.

Study	Year	Region	Models	Key results	Dataset	Key findings	Thalassemia subtypes (alpha major/minor, beta major/minor)	Multiclass detection
Umar et al. ^26^	2025	Pakistan	XGBoost, CNN	99.34% (Acc for alpha thalassemia), 98.10% (Acc for beta thalassemia)	Feature Based (Self Collected 20, 041 records) Feature Based (Public available dataset)	Thalassemia	×	×
Donghua et al.^40^	2023	China	DNN	96% (Acc)	Feature-Based (Self Collected) 8693 records (2014–2021)	Detection	×	×
Shoaib et al.^41^	2023	Pakistan	FL	92.38% (Acc)	Feature-Based (Self Collected) 5066 Patients	Beta Thalassemia Detection	×	×
Rustam et al.^42^	2022	Pakistan	CNN for detection, PCA for feature selection	96.00% (Acc)	Feature-Based (Self Collected) 5066 Patients	Beta Thalassemia Detection	×	×
Ucucu et al.^43^	2022	Turkey	KNN, Naïve Bayes, DT, Boruta Algorithm (Feature selection)	99.00% (Acc)	Feature-Based (Self Collected) 238 Patients (90 Women and 148 Men) (2015 to 2021)	Hemoglobin variants (HbS and HbD)	×	×
Feng et al.^44^	2022	China	RF	91.5% (Acc)	Feature-Based (Self Collected) 1213 Patients. 495 Pregnant (2018–2020)	Alpha Thalassemia Detection	×	×
ER Susanato et al.^45^	2022	Indonesia	Fuzzy Model	Not Mention	Feature-Based (Self-Collected) developed a web-based application	Thalassemia Detection	×	×
Rena et al.^46^	2022	India	Machine Learning Algorithms	86.6% (Acc)	Feature-Based (Self Collected) 1076 Samples	Beta Thalassemia Detection	×	×
Salman et al.^47^	2022	Pakistan	MobilenetV2	95.72% (Acc)	Image Based (Self-Collected in 2 years) 524 Images	Alpha Thalassemia Detection		
Sadiq et al.^48^	2021	Pakistan	Ensemble Learning	93% (Acc)	Feature-Based (Self Collected) 5066 Patients	Beta Thalassemia Detection	×	×
Fu et al.^49^	2021	Taiwan	SVM	0.76 (AUC)	Feature-Based (Self-Collected) 350 Patients (2018–2020)	Thalassemia Detection	×	×
Laengsri et al.^50^	2019	Thailand	RF, KNN, ANN	95.50% (Acc)	Feature-Based (Self Collected) 186 Patients (2014–2016)	Thalassemia Detection	×	×
Monalisha et al.^51^	2018	Thailand	KNN	93.89% (Prec)	Feature-Based (Self Collected) 1500 Samples	Hemoglobin variants Detection	×	×
Farhadi et al.^52^	2018	Tehran	RF, DT	0.21 (Sen) 0.77 (Spec)	Feature-Based (Self Collected) 3489 Cases in 2018	Thalassemia Detection	×	×
Jahangiri et al.^53^	2017	Tehran	DT	0.99 (AUC)	Feature-Based (Self Collected) 144 Patients	Beta Thalassemia	×	×
Kandhro et al.^54^	2017	Pakistan	DT, RF	90% (Spec)	Feature-Based (Self-Collected) 3030 Patients	Alpha and Beta Thalassemia	×	×
Risoluti et al.^55^	2016	Italy	PLS	89.9% (Sen)	Image-Based (Self Collected) 63 Patients	Beta Thalassemia	×	×
Matos et al.^56^	2016	Brazil	Fisher Discriminant	99.3% (Matos Index)	Feature-Based (Self Collected) 185 Patients	Alpha and Beta Thalassemia	×	×
Huang et al.^57^	2015	Taiwan	10 Formulae	89.62% (Sen)	Feature-Based (Self Collected) 877 Patients	Alpha and Beta Thalassemia	×	×
Masala et al.^58^	2013	Italy	KNN, PNN	91% (Spec)	Feature-Based (Self-Collected) 304 Patients	Alpha Thalassemia	×	×
Barnhart Magen et al.^59^	2013	Israel	ANN	0.897 (Sen)	Feature-Based (Self-Collected) 526 Patients	Alpha and Beta Thalassemia	×	×
Janel et al.^60^	2012	France	11 Formulae	93% (Acc)	Feature-Based (Self Collected) 129 Patients	Beta Thalassemia	×	×
Shen et al.^61^	2010	China	12 Formulae	0.947 (AUC)	Feature-Based (Self Collected) 300 Cases	Beta Thalassemia Detection	×	×
Urrechaga et al.^62^	2008	Spain	MDA	87.9% (Acc) (Beta) 83.3% (Acc) (Alpha) 72.1% (Acc) (Mixed)	Feature-based (Self Collected) 250 Patients	Alpha and Beta Thalassemia	×	×
George et al.^63^	2007	Greece	6 Formulae	75.06% (Sen)	Feature-Based (Self Collected) 373 Patients	Beta Thalassemia Detection	×	×
Amendolia et al.^64^	2003	Italy	SVM, KNN, MLP	95% (Spec)	Feature-Based (Self Collected) 304 records	Thalassemia Detection		

It underscores the importance of accurately identifying α thalassemia and β-thalassemia carriers to facilitate early intervention and genetic counselling. The paper discusses the limitations of current prediction models and the need for more effective methods. The objectives of the study are clearly outlined as follows:

- To develop machine learning-based techniques to detect alpha thalassemia and beta thalassemia both major and minor carriers.
- To evaluate the performance of the proposed model using primary performance metrics and compare it with existing approaches.
- To analyze the models in terms of multiclass detection with thalassemia subtypes.

## Dataset

The dataset of the proposed model was collected from the Punjab Thalassemia Prevention Program (PTPP) in Pakistan. Currently, the PTPP is focused on a program to eradicate thalassemia in the country. Its main aim is to perform a diagnostic test to distinguish alpha and beta thalassemia cases associated with HbA. When any type of thalassemia carrier is found, a multi-tier screening process takes place for the next generations of the carrier including the parents. In performing its tests, PTPP carries out around 400,000 in that 1 year. The records in the dataset include 9987 individuals who are alpha thalassemia carriers through HPLC testing; 11,000 beta-thalassemia carriers diagnosed through HPLC testing; 10,060 alpha thalassemia patients identified through CBC testing; and 9981 beta-thalassemia patients diagnosed through the CBC testing. Table 2 depicts the dataset features of CBC and Table 3 depicts the dataset features of HPLC reports.

**Table 2:** CBC dataset features.

Feature	Normal range	Data type
Age	Patients’ age cluster 1. 4–10 2. 11–18 3. 19–26 4. 27–38 5. 39–45	Numeric
Sex	Patients’ gender	Categorical
History	Any family history 0 (No family history) 1 (Family history)	Numeric
HB	Men: 13.8–17.2 g/dl Women: 12.1–15.1 g/dl Children: 11.5–15.5 g/dl	Numeric
PCV	Men: 40–52% Women: 36–48% Children: 35–45%	Numeric
RBC	Men: 4.7–6.1 mcL Women: 4.2–5.4 mcL Children: 4.1–5.5 mcL	Numeric
MCV	Adults: 80–100 fL Children: 73–87 fL	Numeric
MCH	Adults: 27–33 pg Children: 25–31 pg	Numeric
MCHC	Adults: 32–36 g/dL Children: 32–36 g/dL	Numeric
RDW	Adults: 11.5–14.5% Children: 11.5–14.5%	Numeric
WBC	Adults: 4500–11,000 cells/μL Children: 5000–14,500 cells/μL	Numeric
NEUT	Adults: 40–60% of WBC Children: 30–60% of WBC	Numeric
Lymph	Adults: 20–40% of WBC Children: 40–70% of WBC	Numeric
Plt	Adults: 150,000–450,000 plt/μL Children: 150,000–450,000 plt/μL	Numeric
Final finding	Diagnosis of alpha thalassemia or beta thalassemia of both major or minor	Categorical

**Table 3:** HPLC dataset features.

Feature	Normal range	Datatype
Age	Patients’ age cluster 1. 4–10 2. 11–18 3. 19–26 4. 27–38 5. 39–45	Numeric
Sex	Patients’ gender	Categorical
History	Any family history 0 (No family history) 1 (Family history)	Numeric
HB	Men: 13.8–17.2 g/dl Women: 12.1–15.1 g/dl Children: 11.5–15.5 g/dl	Numeric
RBC	Men: 4.7–6.1 mcL Women: 4.2–5.4 mcL Children: 4.1–5.5 mcL	Numeric
HCT	Men: 40–52% Women: 36–48% Children: 35–45%	Numeric
MCV	Adults: 80–100 fL Children: 73–87 fL	Numeric
MCH	Adults: 27–33 pg Children: 25–31 pg	Numeric
MCHC	Adults: 32–36 g/dL Children: 32–36 g/dL	Numeric
RDW	Adults: 11.5–14.5% Children: 11.5–14.5%	Numeric
HbA	Adults: 95–98% Children: Same	Numeric
HbA2	Adults: 2.5–3.5% Children: Same	Numeric
HbF	Adults: Less than 1–2% Children: Same	Numeric
Final finding	Diagnosis of alpha thalassemia or beta thalassemia of both major or minor	Categorical

## Methodology

**Fig. 2:**
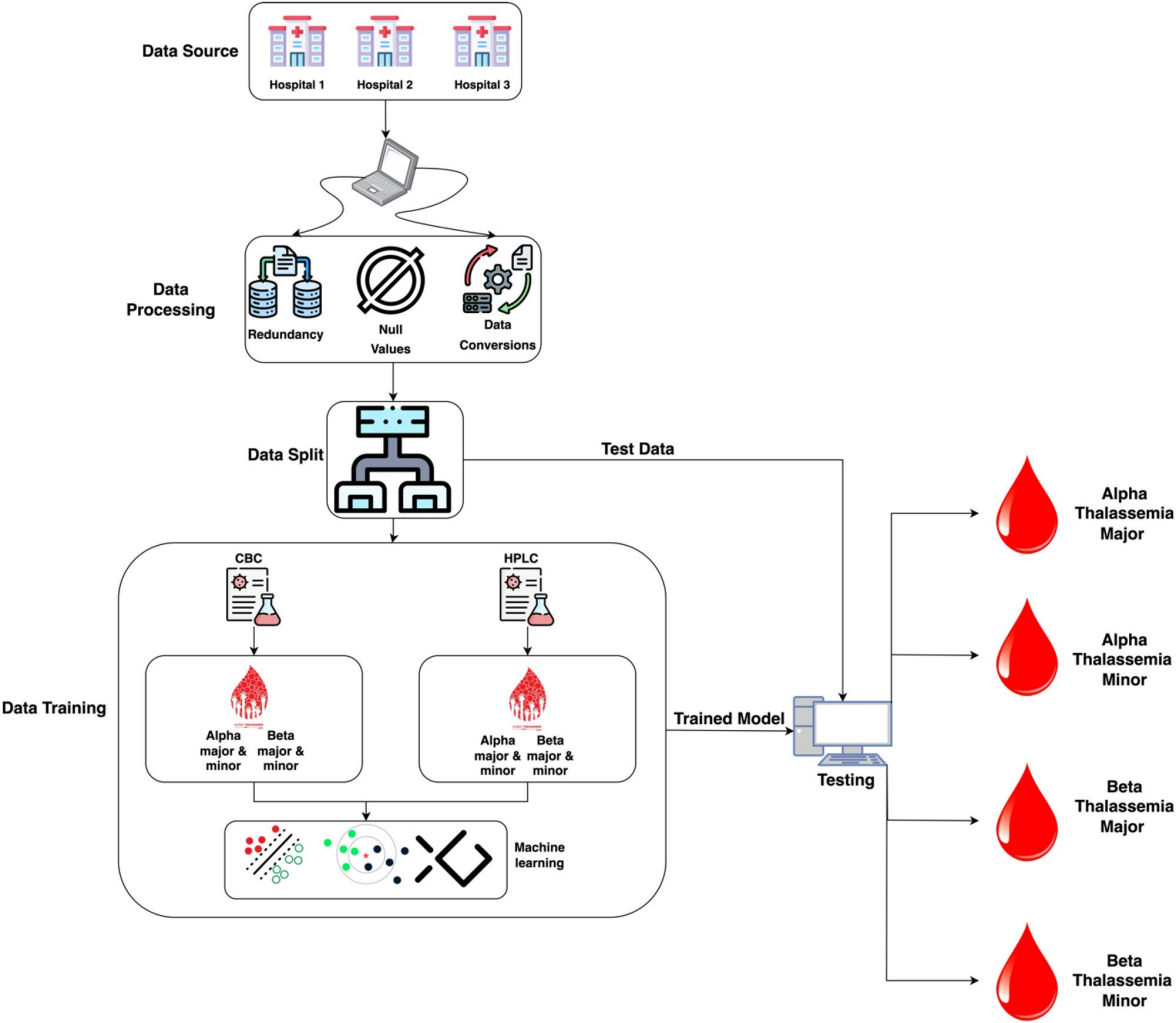
The proposed model for the detection of thalassemia variants using machine learning.

The proposed model used an efficient machine learning approach for identifying carriers of alpha and beta-thalassemia. The proposed model used a MacBook Pro 2017 with 16 GB RAM and 512 GB SSD with MATLAB 2020 for training and testing purposes. Figure 2 depicts the outline of the proposed model and it is explained below.

### Dataset collection

The data will be collected from different hospitals, and it will comprise alpha and beta thalassemia (major and minor) patients, which ensuring dataset diversity. Collection refers to ethical practices and protects patient’s information.

### Data preprocessing

- *Duplicate removal* All duplicates are removed to prevent of different data errors.
- *Handling missing values* There are cases where values must be completely missing, to address that, null values are imputed statistically or excluded.
- *Encoding categorical variables* Some of the types of data are converted to numerical form such as the one-hot encoding for the genetic markers and other categorical data.

### Data splitting

The dataset will be divided into:

- *Training set (70%)* Used for training models.
- *Testing Set (30%)* Used for performance assessment of the built model.

### Model training

Feature Selection: Hence, in this work, nominal data from CBC and HPLC are used to derive distinguishing features.

#### Machine learning models

KNN: Divides cases by measuring the distances between various points, and then sorting them.

SVM: It categorizes them into classes based on the use of optimized hyperplanes.

XGBoost: Uses gradient boosting approach to raise the classification accuracy focusing on the important features.

### Testing and evaluation

#### Testing

The models are then evaluated for the test set.

Assess performance based on all the Thalassemia classes; the major and minor carriers.

#### Performance metrics^65,66^

1 $$\omega_{p} = {\raise0.7ex\hbox{${\vartheta_{p} }$} \!\mathord{\left/ {\vphantom {{\vartheta_{p} } {\psi_{p} }}}\right.\kern-0pt} \!\lower0.7ex\hbox{${\psi_{p} }$}}$$

$\therefore$ $\vartheta$ is for the predicted class, $\psi$ for the true class and $\omega$ represents a true positive class

2 $$\beta_{p} = \mathop \sum \limits_{h = 1}^{3} \left( {{\raise0.7ex\hbox{${\vartheta_{p} }$} \!\mathord{\left/ {\vphantom {{\vartheta_{p} } {\psi_{h \ne p} }}}\right.\kern-0pt} \!\lower0.7ex\hbox{${\psi_{h \ne p} }$}}} \right)$$

$\therefore$ $\beta$ represents the true negative class, the sum of all three predicted classes

3 $$\xi_{p} = \mathop \sum \limits_{h = 1}^{3} \left( {{\raise0.7ex\hbox{${\vartheta_{h \ne p} }$} \!\mathord{\left/ {\vphantom {{\vartheta_{h \ne p} } {\psi_{p} }}}\right.\kern-0pt} \!\lower0.7ex\hbox{${\psi_{p} }$}}} \right)$$

$\therefore$ $\xi$ represents the false positive class, the sum of all three predicted classes

4 $$\gamma_{p} = \mathop \sum \limits_{h = 1}^{3} \left( {{\raise0.7ex\hbox{${\vartheta_{h \ne p} }$} \!\mathord{\left/ {\vphantom {{\vartheta_{h \ne p} } {\psi_{h \ne p} }}}\right.\kern-0pt} \!\lower0.7ex\hbox{${\psi_{h \ne p} }$}}} \right)$$

$\therefore$ $\gamma$ represents false-negative class, the sum of all three predicted classes

5 $${\text{Accuracy}} = \frac{{\omega_{p} + \beta_{p} }}{{\omega_{p} + \beta_{p} + \xi_{p} + \gamma_{p} }} * 100$$

6 $${\text{Misclassification Rate}} = 100 - \left( {\frac{{\omega_{p} + \beta_{p} }}{{\omega_{p} + \beta_{p} + \xi_{p} + \gamma_{p} }}*100} \right)$$

7 $${\text{Sensitivity}} = \frac{{\omega_{p} }}{{\omega_{p} + \gamma_{p} }} * 100$$

8 $${\text{Specificity}} = \frac{{\beta_{p} }}{{\beta_{p} + \xi_{p} }} * 100$$

9 $${\text{F}}1 - {\text{Score}} = \frac{{2 \omega_{p} }}{{2 \omega_{p} + \xi_{p} + \gamma_{p} }} * 100$$

10 $${\text{False Positive Rate}} = 100 - \left( {\frac{{\beta_{p} }}{{\beta_{p} + \xi_{p} }}*100} \right)$$

11 $${\text{False Negative Rate}} = 100 - \left( {\frac{{\omega_{p} }}{{\omega_{p} + \gamma_{p} }}*100} \right)$$

## Machine learning models

All machine learning models are simulated by the proposed model stated below.

### SVM

SVM are binary classifiers. For multi-class classification, ECOC decomposes the multi-class problem into multiple binary problems. Each binary problem is handled by a separate SVM. The results from all binary SVMs are then combined to predict the final class.

Error-Correcting Output Codes (ECOC).

ECOC creates a coding matrix $M \epsilon \left\{ { - 1,0, 1} \right\}^{CxK}$, where:

$C$: Number of classes.

$K$: Number of binary classifiers.

$M_{ik}$: Specifies the association of class $i$ with the $k - th$ binary classifier:

1: Positive class for classifier $k$.

− 1: Negative class for classifier $k$.

O: Class $i$ is not considered in classifier $k$.

#### SVM training: finding the optimal hyper plane

For each binary problem in ECOC, an SVM is trained to find the hyperplane that maximizes the margin between the two classes.

SVM Optimization Problem.

The optimization problem for SVM is:

$${}_{w,b,\xi }^{{{\text{min}}}} \frac{1}{2}\parallel w\parallel^{2} + C\mathop \sum \limits_{i = 1}^{n} \varepsilon_{i}$$

Subject to:

$$y_{i} \left( {w^{T} \phi \left( {x_{i} } \right) + b} \right) \pm 1 - \xi_{i} ,\xi_{i} \pm 0,\forall_{i}$$

$w$: Weight vector defining the hyperplane. $b$: Bias term. $\phi \left( {x_{i} } \right)$: Feature mapping to a higher-dimensional space. $C$: Box constraint (penalty for misclassifications). $\xi_{i}$: Slack variable for sample $i$, representing its margin violation.

#### Polynomial kernel

In the code, a polynomial kernel is used to map features into a higher-dimensional space.

$$K\left( {x_{i} ,x_{j} } \right) = \left( {\gamma x_{i}^{T} x_{j} + r} \right)^{d}$$

$x_{i} ,x_{j}$: Input feature vectors. $\gamma$: Kernel scale (set to “auto” in the code). $r$: Coefficient term. $d$: Degree of the polynomial (default is 3).

#### Dual Formulation of SVM

SVM is typically solved in its dual formulation using Lagrange multipliers:

$${}_{ \propto }^{max} \mathop \sum \limits_{i = 1}^{n} \propto_{i} - \frac{1}{2}\mathop \sum \limits_{i = 1}^{n} \mathop \sum \limits_{j = 1}^{n} \propto_{i} \propto_{j} y_{i} y_{j} K\left( {x_{i} ,x_{j} } \right)$$

Subject to

$$\mathop \sum \limits_{i = 1}^{n} \propto_{i} y_{i} = 0,0 \le \propto_{i} \le C,\forall_{i}$$

$\propto_{i}$: Lagrange multipliers. $K\left( {x_{i} ,x_{j} } \right)$: Kernel function.

#### Multi-class prediction

For prediction, ECOC combines the outputs from all binary classifiers:

1. Compute the signed decision function for each binary SVM:
  $$f_{k} \left( x \right) = \mathop \sum \limits_{i = 1}^{n} \propto_{i} y_{i} K\left( {x_{i} ,x} \right) + b_{k}$$
2. Convert decision function values into class-specific scores:
  $$D_{i,k} = sign\left( {f_{k} \left( x \right)} \right)$$
3. Compute $D_{i,k}$ with the ECOC matrix $M$:
  $$\hat{y} = {}_{i}^{argmin} \mathop \sum \limits_{k = 1}^{K} \parallel \left[ {M_{i,k} \ne D_{i,k} } \right]$$

$D_{i,k}$: Decision value for class $i$ and classifier $k$. $\parallel$: Indicator function (1 if true, O otherwise). $\hat{y}$: Predicted class.

#### Hyperparameter details

The code uses these hyperparameters:

- *Kernel function* Polynomial kernel.
- *Kernel scale* Automatically adjusts $\gamma$ in the kernel function.
- *Box constraint* ($C$) Regularization parameter controlling the trade-off between margin width and misclassification penalty.

#### Feature importance in SVM

SVM does not provide explicit feature importance, but approximate importance can be derived using the weight vector in the primal form:

$$IMPORTANCE\left( {f_{j} } \right) = \omega_{j}^{2}$$

$\omega_{j}^{2}$: Weight of feature $j$.

The Pseudocode of SVM is stated below.
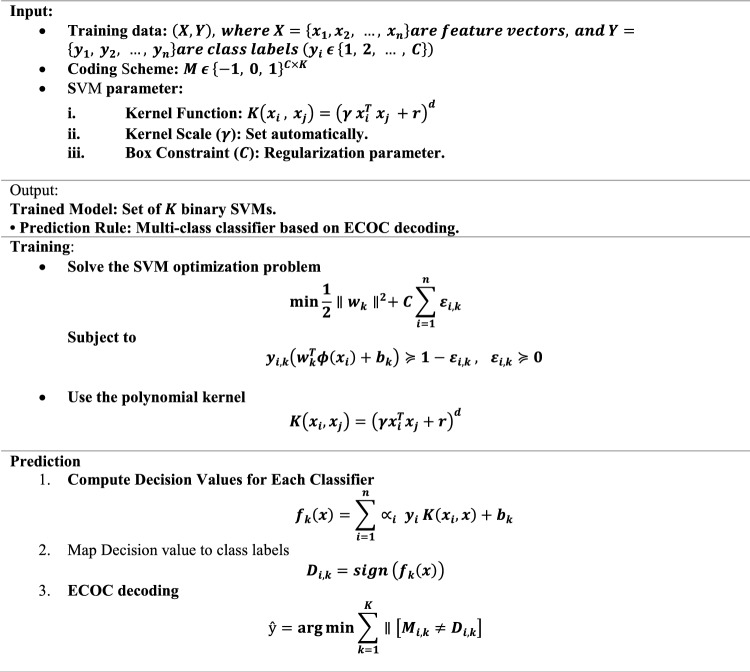


### k-NN

k-NN is a non-parametric, instance-based algorithm. It predicts the class of a query point by evaluating the majority class among its k-nearest neighbors in the feature space.

Core Steps in k-NN:

- Compute distances between the query point and all training points.
- Identify the k-nearest neighbors.
- Predict the majority class among the neighbors.

#### Parameter in the code

- Num neighbors $${\text{K}}$$ = 2.
  The model considers the 2 nearest neighbors for each prediction.
- Distance metric
  $$d\left( {x_{i} ,x_{j} } \right) = \left( {\mathop \sum \limits_{l = 0}^{p} \left| {xi,l - xj,l} \right|^{q} } \right)^{\frac{1}{q}}$$
  $x_{i} ,x_{j}$: Feature vectors of the two points. $q$: Order of the Minkowski distance. $q = 2$: Euclidean distance. $q = 1$: Manhattan distance.
- Standardization
  Features are standardized to ensure equal contribution to distance metrics:
  $$x^{\prime} = \frac{x - \mu }{\sigma }$$
  $x$: Original feature value. $\mu$: Mean of the feature. $\sigma$: Standard deviation of the feature.

#### Prediction work flow

- Distance computation.
  For each query point $x_{query}$, compute the distance to every training point $x_{i}$:
  $$d\left( {x_{query} ,x_{i} } \right) = \left( {\mathop \sum \limits_{l = 0}^{p} \left| {x_{query,l} - x_{i,l} } \right|^{q} } \right)^{\frac{1}{q}}$$
- Find nearest neighbors
  Sort the distances and select the $k = 2$ smallest distances. Let $N_{k} \left( {x_{query} } \right)$ denote the indices of these neighbors.
- Assign class
  $$\hat{y} = {}_{c}^{{\arg {\text{max}}}} \mathop \sum \limits_{{i\epsilon N_{k} \left( {x_{query} } \right)}} \parallel \left( {y_{i} = c} \right)$$
  $\hat{y}$: Predicted class label. $c$: A candidate class. $N_{k} \left( {x_{query} } \right)$: Indices of the k-nearest neighbors. $\parallel \left( {y_{i} = c} \right)$: Indicator function (1 if $y_{i} = c$, otherwise 0).

#### Multi-class majority voting

In multi-class k-NN, the neighbors can belong to multiple classes. The algorithm predicts the class with the highest vote count. In the case of ties, MATLAB typically resolves them randomly or based on internal rules.

$$\hat{y} = {}_{c\epsilon C}^{{\arg {\text{max}}}} \mathop \sum \limits_{{i\epsilon N_{k} \left( {x_{query} } \right)}} \parallel \left( {y_{i} = c} \right)$$

$C$: Set of all possible classes. $\mathop \sum \limits_{{i\epsilon N_{k} \left( {x_{query} } \right)}} \parallel \left( {y_{i} = c} \right)$: Count of neighbors belonging to class $c$.

#### Key mathematical concepts

- Minkowski distance.
  The Minkowski distance generalizes distance metrics with a parameter $q$:
  $$d\left( {x_{i} ,x_{j} } \right) = \left( {\mathop \sum \limits_{l = 1}^{p} \left| {x_{i,l} - x_{j,l} } \right|^{q} } \right)^{\frac{1}{q}}$$
  $q = 2$: Euclidean distance. $q = 1$: Manhattan distance.
- Feature standardization.
  Standardizing features ensures uniform scaling:
  $$x^{\prime } = \frac{x - \mu }{\sigma }$$
  Ensures each feature contributes equally to distance computation.
- Majority voting.
  For a multi-class problem:
  $$\hat{y} = {}_{c}^{{\arg {\text{max}}}} \mathop \sum \limits_{{i\epsilon N_{k} \left( {x_{query} } \right)}} \parallel \left( {y_{i} = c} \right)$$
- Exhaustive search
  For exhaustive search:
  1. Compute distances for all points:
  $$D = \left\{ {d\left( {x_{query} ,x_{1} } \right),d\left( {x_{query} ,x_{2} } \right), \ldots ,d\left( {x_{query} ,x_{n} } \right)} \right\}$$
  2. Sort $D$ and select the $k$ smallest distances

The Pseudocode of KNN is stated below.
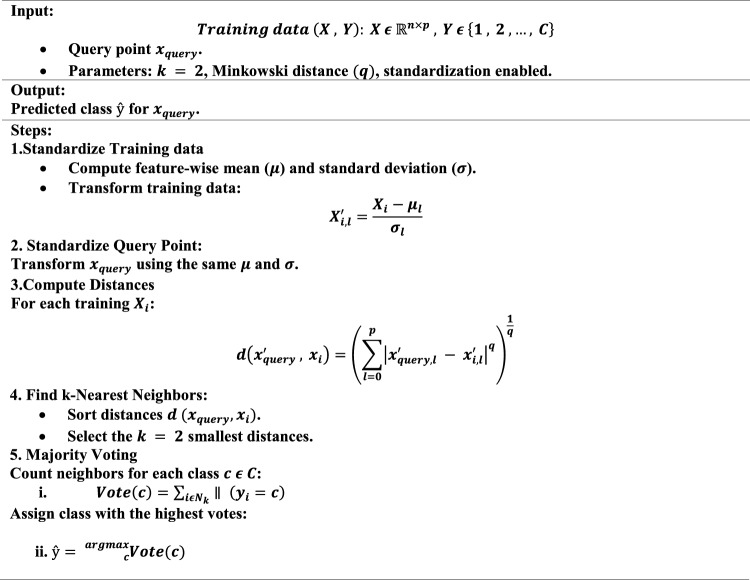


### XGBoost

AdaBoostM2 extends binary AdaBoost to handle multi-class classification by introducing a pseudo-loss and adjusting the weights of samples across multiple classes. Model iterative equation mention below.

$$F_{m} \left( x \right) = F_{m - 1} \left( x \right) + \eta \cdot h_{m} \left( x \right)$$

$F_{m} \left( x \right)$: Prediction of the ensemble model after m-th iteration. $F_{m - 1} \left( x \right)$: Prediction of the ensemble after (m − 1)-th iteration. $\eta$: Learning rate controlling the contribution of each weak learner. $h_{m} \left( x \right)$: Weak learner (e.g., decision tree) fitted at the m-th iteration.

#### Pseudo-loss for multi-class boosting

AdaBoostM2 minimizes the pseudo-loss, which generalizes binary classification loss to multi-class settings by considering the incorrect prediction probabilities across all classes.

$$PseudoLoss\left( {h_{m} } \right) = \mathop \sum \limits_{i = 1}^{n} \mathop \sum \limits_{k\ddag yi} W_{i} .\parallel \left[ {h_{m} \left( {x_{i} } \right) = k} \right] \cdot P_{i,k}$$

$W_{i}$: Weight of the i-th training sample. $P_{i,k}$: Probability that 2; belongs to class k, calculated based on previous iterations. $h_{m} \left( {x_{i} } \right)$: Predicted class of a; by the weak learner at iteration m. $\parallel \left[ . \right]$: Indicator function (1 if the condition is true, otherwise 0). $yi$: True class label of $x_{i}$.

#### Weight update for multi-class sample

The weights of samples are adjusted based on their contribution to the pseudo-loss. Misclassified samples or classes receive higher weights to emphasize learning.

$$W_{i}^{{\left( {m + 1} \right)}} = W_{i}^{\left( m \right)} \cdot \exp \left( { \propto_{m} \cdot \mathop \sum \limits_{{k \ne y_{i} }} P_{i,k} \cdot \parallel \left[ {h_{m} \left( {x_{i} } \right) = k} \right]} \right)$$

$\propto_{m}$: Weight of the m-th weak learner, reflecting its accuracy

$$\propto_{m} = \frac{1}{2}\ln \left( {\frac{{1 - PseudoLoss\left( {h_{m} } \right)}}{{PseudoLoss\left( {h_{m} } \right)}}} \right)$$

#### Final prediction for multi-class

The ensemble model aggregates predictions across all iterations and assigns the label with the highest cumulative score.

$$\hat{y} = {}_{k}^{{\arg {\text{max}}}} \mathop \sum \limits_{m = 1}^{M} \propto_{m} \cdot \parallel \left[ {h_{m} \left( x \right) = k} \right]$$

$\hat{y}$: Predicted class label. $M$: Total number of iterations. $\propto_{m}$: Weight of the m-th weak learner. $\parallel \left[ {h_{m} \left( x \right) = k} \right]$: Indicator for whether the m-th learner predicts class k.

#### Residual calculation for multi-class

Residuals for multi-class boosting represent the error probability for the predicted class versus the true class.

$$r_{i,k}^{\left( m \right)} = \left\{ {\begin{array}{*{20}c} {P_{i,k} } & {if\;k = y_{i} } \\ {P_{i,k} } & {if\;k \ne y_{i} } \\ \end{array} } \right.$$

$r_{i,k}^{\left( m \right)}$: Residual for sample $i$ and class $k$ at iteration $m$.

#### Feature importance in multi-class

Feature importance measures the contribution of each feature to the splits in all weak learners.

$$I\left( {f_{k} } \right) = \mathop \sum \limits_{t = 1}^{T} \mathop \sum \limits_{c = 1}^{C} \Delta G_{t} \left( {f_{k} ,c} \right)$$

$I\left( {f_{k} } \right)$: Importance of feature $f_{k}$. $T$: Total number of splits across all trees. $c$: Total number of classes. $\Delta G_{t} \left( {f_{k} ,c} \right)$: Reduction in impurity for feature $f_{k}$ for class $c$ at split $T$.

#### Probability prediction for multi-class

For multi-class problems, the output of the ensemble is converted into class probabilities.

$$p\left( {y = k{|}x} \right) = \frac{{\exp \left( {\mathop \sum \nolimits_{m = 1}^{M} \propto_{m} \cdot \parallel \left[ {h_{m} \left( x \right) = k} \right]} \right)}}{{\mathop \sum \nolimits_{j = 1}^{k} \exp \left( {\mathop \sum \nolimits_{m = 1}^{M} \propto_{m} \cdot \parallel \left[ {h_{m} \left( x \right) = j} \right]} \right)}}$$

$p\left( {y = k{|}x} \right)$: Probability of class $k$ given input $x$. $k$: Total number of classes.

#### Regularization in multi-class gradient boosting

Regularization controls overfitting by reducing the learning rate or limiting the complexity of weak learners.

$$F_{m} \left( x \right) = F_{m - 1} \left( x \right) + \eta \cdot \min \left( {\left| {\frac{{r_{i,k}^{\left( m \right)} }}{{W_{i}^{\left( m \right)} }}} \right|,\tau } \right)$$

$\tau$: Regularization threshold for residuals.

Pseudocode of XGBoost is stated below.
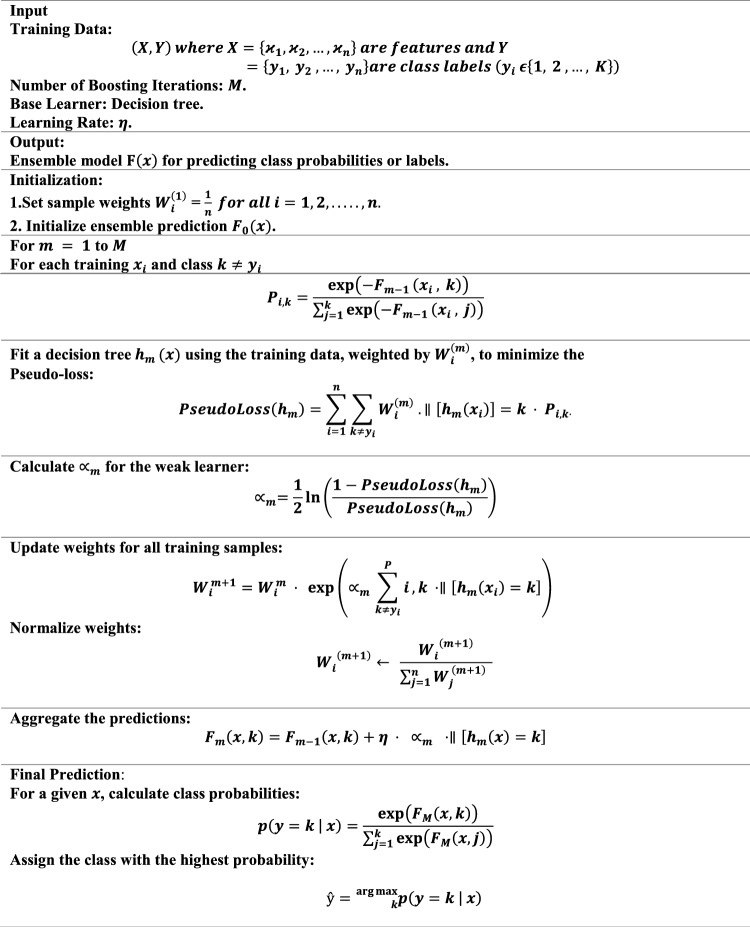


## Results

The classifier model trainings were conducted on a 2017 MacBook Pro, Core i5, and high-performance graphics. It undergoes numerous tests to assess the best classifiers for various conditions such as alpha thalassemia major and minor, and beta thalassemia major and minor. These assessments are evaluated using both HPLC and CBC data.

**Fig. 3:**
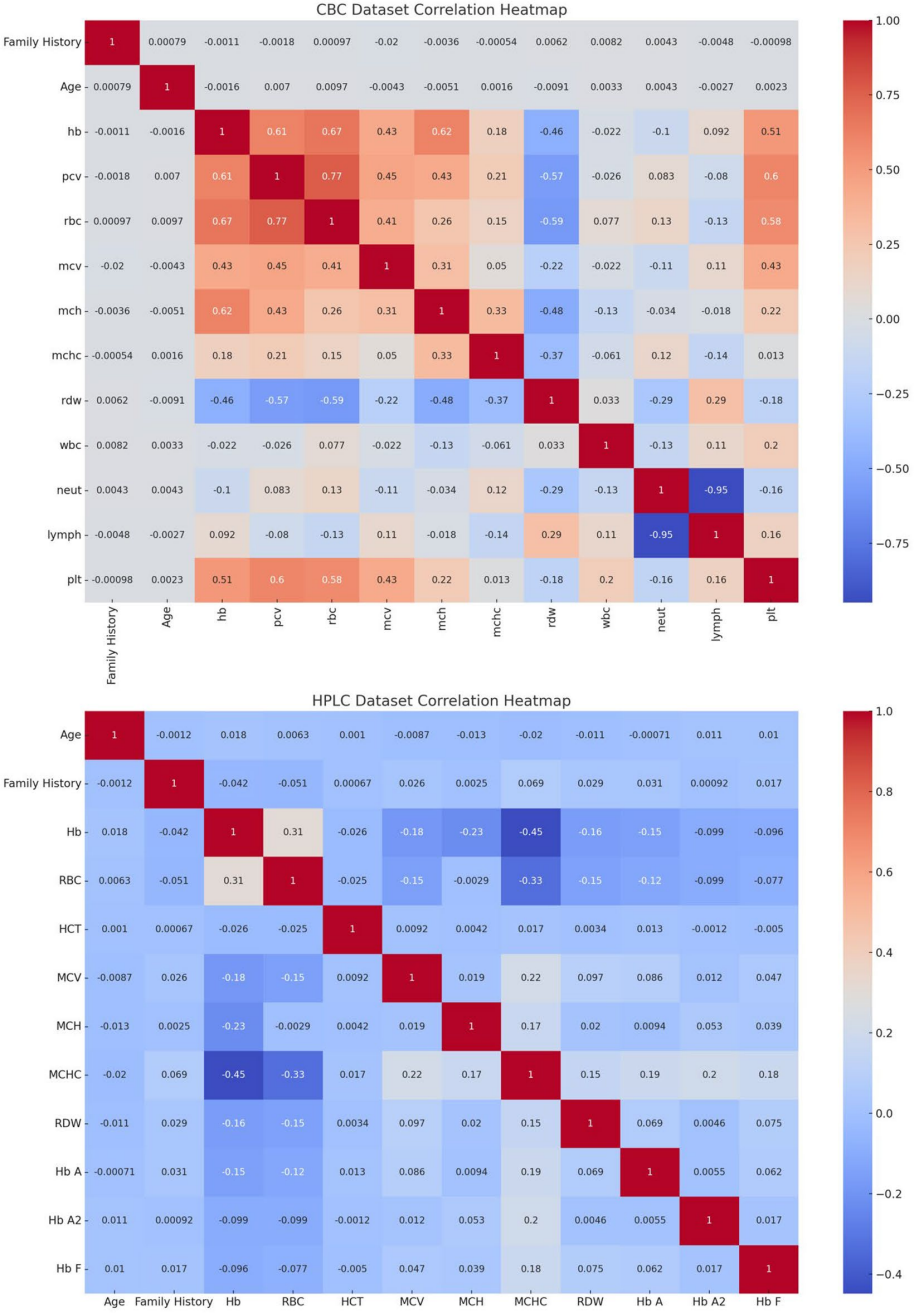
Correlation heatmap visualization of CBC and HPLC dataset.

Figure 3 shows the correlation heatmap of CBC and HPLC dataset so, CBC heatmap shows the strength levels through shading intensity where darkness indicates stronger connections between positive or negative values. It makes clinical sense why the blood factors hb, pcv and rbc exhibit substantial positive relationships since their changes align together. Two CBC indicators namely wbc and plt demonstrate weaker associations with other blood parameters but show no significant relationship. Analyzing blood factor connections enables the identification of how blood components behave across the alpha major, beta major, beta minor and alpha minor types of thalassemia. The analysis reveals similar and contrasting patterns in the HPLC dataset heatmap between basic hematological features and the quantity levels of Hb A, Hb A2, and Hb F. The medical correlation understanding between Hb A and Hb F demonstrates negative strength especially when patients have beta thalassemia major. The distinct relations between Hb A2 help identify minor and major versions of thalassemia. The pattern recognition from HPLC results enables proper identification of patients with beta major, beta minor, alpha major and Alpha Minor.

Table 4 shows the training set for the K-Nearest Neighbours (KNN) model using Complete Blood Count (CBC) data. The proposed approach has greater accuracy in distinguishing between alpha thalassemia major, alpha thalassemia minor, beta thalassemia major, and beta thalassemia minor. In the instance of Alpha Major, it attained an accuracy of identifying 1985 cases as belonging to this group with no mistake in categorizing them as belonging to any other category. Additionally, the model displayed great accuracy in discriminating between the four Thalassemia types. Alpha Major accurately categorized 1985 instances, with no misclassifications to other categories. Similarly, Beta Major has 3527 valid categorization instances and no aspects of misunderstanding with other classes. Generate needed templates from the formula Beta Minor was mostly correct with 3349 Correctly Classified Instances, however, there was some misunderstanding with Alpha Major: 111 Misclassified Instances. The 4992 examples recognized for Alpha Minor, with just 23 misclassified as Alpha Major, demonstrate an excellent level of validation accuracy. These findings indicate that the KNN performed well in distinguishing classes in the training data, with only small mistakes that had little effect on the outcomes.

**Table 4:** Multiclass training confusion matric of KNN to detect alpha major thalassemia, alpha minor thalassemia, beta major thalassemia and beta minor thalassemia using CBC.

Classes	Alpha major	Beta major	Beta minor	Alpha minor
Alpha major	1985	0	0	42
Beta major	0	3527	0	0
Beta minor	0	111	3349	0
Alpha minor	23	0	0	4992

As shown in Table 5, when the model was tested on the KNN model for detecting Thalassemia types using CBC data, it achieved high accuracy with few misclassifications. For Alpha Major, precision was achieved with the ability to classify 828 relevant examples and the error of categorizing 33 as Alpha Minor. Beta Major was likewise trustworthy, with 1444 correctly identified instances; however, 19 cases were misclassified as Beta Minor, indicating some differentiation between these two categories. Beta Minor was right in 1439 occurrences across all test sets, with 92 instances classed as Beta Major. Alpha Minor showed a high level of accuracy, accurately classifying 2134 instances whereas Alpha Major mistakenly identified 23 of them. These findings demonstrate that, while the model’s accuracy remains high during testing, minor misclassifications indicate areas for improvement, especially the classification of Beta Minor.

**Table 5:** Multiclass testing confusion matric of KNN to detect alpha major thalassemia, alpha minor thalassemia, beta major thalassemia and beta minor thalassemia using CBC.

Classes	Alpha major	Beta major	Beta minor	Alpha minor
Alpha major	828	0	0	33
Beta major	0	1444	19	0
Beta minor	0	92	1439	0
Alpha minor	23	0	0	2134

As shown in Table 6, the XGBoost model trained on the CBC data from the current study achieved good accuracy across all classes. While evaluating the accuracy, Alpha Major was successfully identified 2016 times out of 2019 times, with only three incorrect classifications. In the case of Beta Major, it correctly identified 3415 while incorrectly classified 56 as Beta Minor. Beta Minor displayed remarkable classification accuracy and efficiency, with 3463 cases properly identified and just 55 instances misclassified. Alpha Minor accurately identified 4976 persons but misclassified 45 of them as belonging to Alpha Major. As a result, XGBoost operates with high accuracy on training data and is somewhat more efficient in eliminating Beta Minor misclassifications than KNN.

**Table 6:** Multiclass training confusion matric of XGBOOST to detect alpha major thalassemia, alpha minor thalassemia, beta major thalassemia and beta minor thalassemia using CBC.

Classes	Alpha major	Beta major	Beta minor	Alpha minor
Alpha major	2016	0	0	3
Beta major	0	3415	56	0
Beta minor	0	55	3463	0
Alpha minor	45	0	0	4976

Table 7 also shows testing data to illustrate XGBoost’s ability to diagnose Thalassemia types using CBC with low misclassification. Despite only one inaccurate classification, Alpha Major had a total of 868 right classifications over the fifteen instances utilized in the experiment. Beta Major was likewise accurate, properly classifying 1489 cases; however, 30 of these were categorized as Beta Minor. In general, the Beta Minor method proved quite accurate, properly classifying 1443 cases while misclassifying 30 cases as Beta Major. As a result, Alpha Minor had a high sensitivity for 2129 correctly identified examples and 22 occasions where the classifier wrongly classified them. This shows that XGBoost has consistently low misclassification rates and is more stable throughout the testing period than KNN, particularly for Beta Major and Beta Minor classes.

**Table 7:** Multiclass testing confusion matric of XGBOOST to detect alpha major thalassemia, alpha minor thalassemia, beta major thalassemia and beta minor thalassemia using CBC.

Classes	Alpha major	Beta major	Beta minor	Alpha minor
Alpha major	868	0	0	1
Beta major	0	1489	30	0
Beta minor	0	30	1443	0
Alpha minor	22	0	0	2129

Table 8 shows that classification SVM demonstrated high accuracy on the CBC training set. In the Alpha Major classification method, 1996 candidates were accurately identified, with just one being misclassified as Alpha Minor. Beta Major demonstrated efficiency with 3442 valid classifications and 62 instances of Beta Minor misdiagnosis. Bet Minor successfully identified 3466 samples but misclassified 57. Alpha Minor had the most correctly identified instances, with 4918, while 40 were misclassified as Alpha Major. SVM’s outcomes are visible in the effective division of classes on the training set, as well as slight gains in limiting Beta’s slight misclassification over previous models.

**Table 8:** Multiclass training confusion matric of SVM to detect alpha major thalassemia, alpha minor thalassemia, beta major thalassemia and beta minor thalassemia using CBC.

Classes	Alpha major	Beta major	Beta minor	Alpha minor
Alpha major	1996	0	0	1
Beta major	0	3442	62	0
Beta minor	0	57	3466	0
Alpha minor	40	0	0	4918

Table 9 shows that the SVM model performed similarly to the CBC testing data, maintaining comparable patterns of correctness. In the instance of Alpha Major, the model attained 821 right classifications, with only two wrong classifications as Alpha Minor. The decision-making accuracy at Beta Major was 1462, with 24 occurrences of Beta Minor misclassification. Beta Minor has a rather high level of precision, with 1439 valid classifications and 29 misclassifications. Alpha Minor remained constant, with 2181 correct classifications and 26 incorrect ones. The improvement is minor, as seen by SVM’s identical performance in testing and training data, with no major misclassifications in Beta Minor differentiation.

**Table 9:** Multiclass testing confusion matric of SVM to detect alpha major thalassemia, alpha minor thalassemia, beta major thalassemia and beta minor thalassemia using CBC.

Classes	Alpha major	Beta major	Beta minor	Alpha minor
Alpha major	821	0	0	2
Beta major	0	1462	24	0
Beta minor	0	29	1439	0
Alpha minor	26	0	0	2181

Using HPLC data, Table 10 depicts the efficiency of KNN training sets. There were 5029 instances accurately categorized as Beta Major, and 42 cases as Alpha Minor. Beta Minor received 2455 correct classifications; however, 199 instances were categorized as Beta Major. Alpha Major correctly identified nearly all of the 2473 instances. The researchers also trained Alpha Minor, which recorded 4259 valid matches while 275 were incorrectly assigned to other classes. KNN’s high accuracy on HPLC data while learning makes it a strong choice for Beta Minor classification with minimal labor.

**Table 10:** Multiclass training confusion matric of KNN to detect alpha major thalassemia, alpha minor thalassemia, beta major thalassemia and beta minor thalassemia using HPLC.

Classes	Beta major	Beta minor	Alpha major	Alpha minor
Beta major	5029	0	0	42
Beta minor	199	2455	1	0
Alpha major	0	0	2473	0
Alpha minor	23	12	240	4259

Table 11 shows that, despite minor misclassification, the KNN model testing accuracy with HPLC data remained consistent. The proposed model was able to accurately capture the type of Beta Major in 2091 samples, however 33 were categorized incorrectly. There were 1071 valid classifications, with 121 instances categorized as Beta Major but really Beta Minor. On the Alpha Major spectrum, 934 cases were correctly identified, whereas 40 cases were incorrectly tagged as Alpha Minor. Alpha Minor has 1808 accurate and 198 misclassificationss, which were separated into several classes to manage branch length. This is good, as seen by the model’s outstanding testing performance with HPLC, although Beta Minor may benefit from increased precision.

**Table 11:** Multiclass testing confusion matric of KNN to detect alpha major thalassemia, alpha minor thalassemia, beta major thalassemia and beta minor thalassemia Using HPLC.

Classes	Beta major	Beta minor	Alpha major	Alpha minor
Beta major	2091	28	2	3
Beta minor	121	1071	0	0
Alpha major	2	0	934	38
Alpha minor	23	8	167	1808

On the HPLC training set, the XGBoost model produced an impressive result demonstrating classification accuracy. Table 12 shows that Beta Major was accurately categorized in 4964 of the reported instances, with 91 incorrect predictions. Beta Minor received a high accuracy classification of 2550, however, Alpha Minor was slightly puzzled at 109. Alpha Major consisted of 2270 accurate classifications and had small misclassifications involving Beta Minor. After analyzing the proposal, Alpha Minor supplied 4471 valid classifications, whereas 121 were misclassified. XGBoost maintains excellent accuracy and low error rates on training data utilizing HPLC in classes.

**Table 12:** Multiclass training confusion matric of XGBOOST to detect alpha major thalassemia, alpha minor thalassemia, beta major thalassemia and beta minor thalassemia Using HPLC.

Classes	Beta major	Beta minor	Alpha major	Alpha minor
Beta major	4964	87	0	4
Beta minor	105	2550	0	5
Alpha major	1	4	2270	109
Alpha minor	13	7	101	4471

XGBoost showed great accuracy when utilizing the HPLC testing data in Table 13. Beta Major achieved 2058 correct classifications, with just 40 incorrect instances. In Beta Minor, all cases were correctly identified 1123, but 62 were misclassified as Beta Major. This was demonstrated by Alpha Major, which had 1031 valid classifications and 32 incorrectly classed as Alpha Minor. When evaluated, Alpha Minor successfully identified 1900 cases, while the remaining 48 were classified as minors or alphas, although wrongly. HPLC also shows that the model maintains consistent performance across test iterations.

**Table 13:** Multiclass testing confusion matric of XGBOOST to detect alpha major thalassemia, alpha minor thalassemia, beta major thalassemia and beta minor thalassemia using HPLC.

Classes	Beta major	Beta minor	Alpha major	Alpha minor
Beta major	2058	40	0	0
Beta minor	62	1123	0	2
Alpha major	2	2	1031	28
Alpha minor	4	2	42	1900

**Fig. 4:**
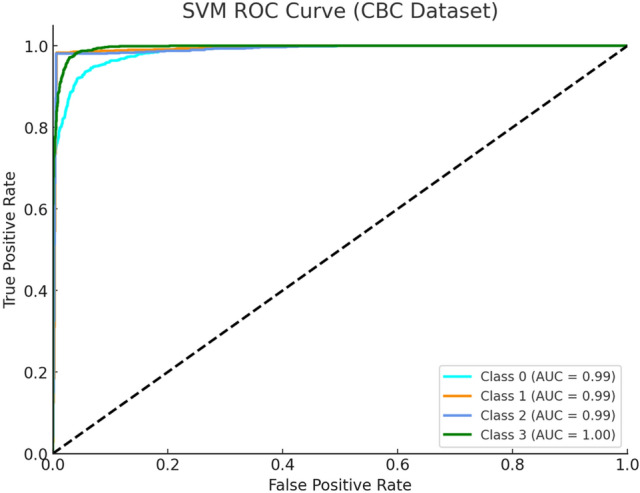
SVM ROC Curve (CBC Dataset).

Figure 4 depicts the SVM ROC curve and analyses CBC data to show how the model identifies among Alpha Major (Class 0), Beta Major (Class 1), Beta Minor (Class 2) and Alpha Minor (Class 3) cases. The SVM model creates distinct clusters with special separation clarity between Beta Major and Beta Minor diagnosis cases. SVM uses a polynomial kernel which produces curves showing strong true positive performance while maintaining low false positive rates in almost all classes thus showing effectiveness for modeling complex haematological decision boundaries.

**Fig. 5:**
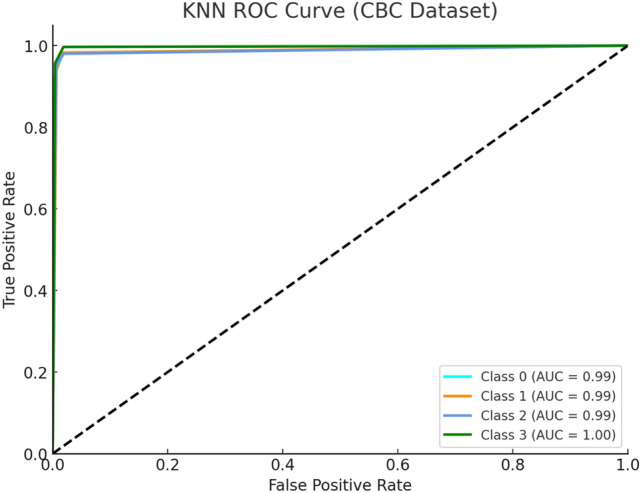
KNN ROC Curve (CBC Dataset).

Figure 5 depicts the KNN ROC curve produced high outcomes on the CBC dataset, particularly for Alpha Major (Class 0) and Beta Major (Class 1). The AUC values of Alpha Minor decrease to lower levels compared to other classes because of poor class separability in KNN analysis. KNN performance fluctuates because it depends on local data density which changes according to feature scaling and class proximity in the CBC profile.

**Fig. 6:**
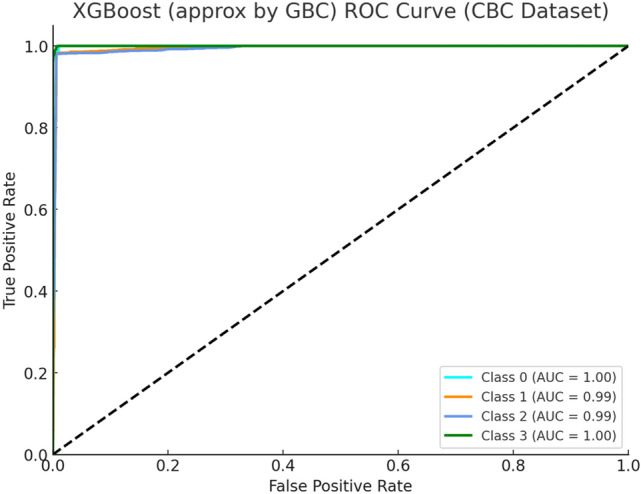
XGBoost ROC Curve (CBC Dataset).

Figure 6 depicts ROC curve analysis that demonstrates superb separation capability between the four thalassemia types by the XGBoost model which was performed on the CBC dataset. The AUC values demonstrate outstanding class separation by reaching almost perfect scores when classifying major conditions such as Alpha Major and Beta Major. The model effectively captures nonlinear patterns between features due to its capabilities with structured clinical data.

Table 14 further shows that on the training data for SVM, high accuracy beta classification was achieved, with 4922 properly categorized and 99 labelled as Beta Minor. Beta Minor has 2612 correct classifications, including 95 actuals that were misclassified as Alpha Major owing to a minor mistake. For Alpha Major, there were 2300 correct diagnoses, whereas only 107 specimens were incorrectly categorized for Beta Minor. Alpha Minor has 4454 valid classifications and 101 incorrect ones. They suggest that when applying SVM, classes are well-distinguished on training data, and the model has good class precision.

**Table 14:** Multiclass training confusion matric of SVM to detect alpha major thalassemia, alpha minor thalassemia, beta major thalassemia and beta minor thalassemia Using HPLC.

Classes	Beta major	Beta minor	Alpha major	Alpha minor
Beta major	4922	93	0	6
Beta minor	81	2612	0	14
Alpha major	2	0	2300	105
Alpha minor	4	1	97	4454

**Fig. 7:**
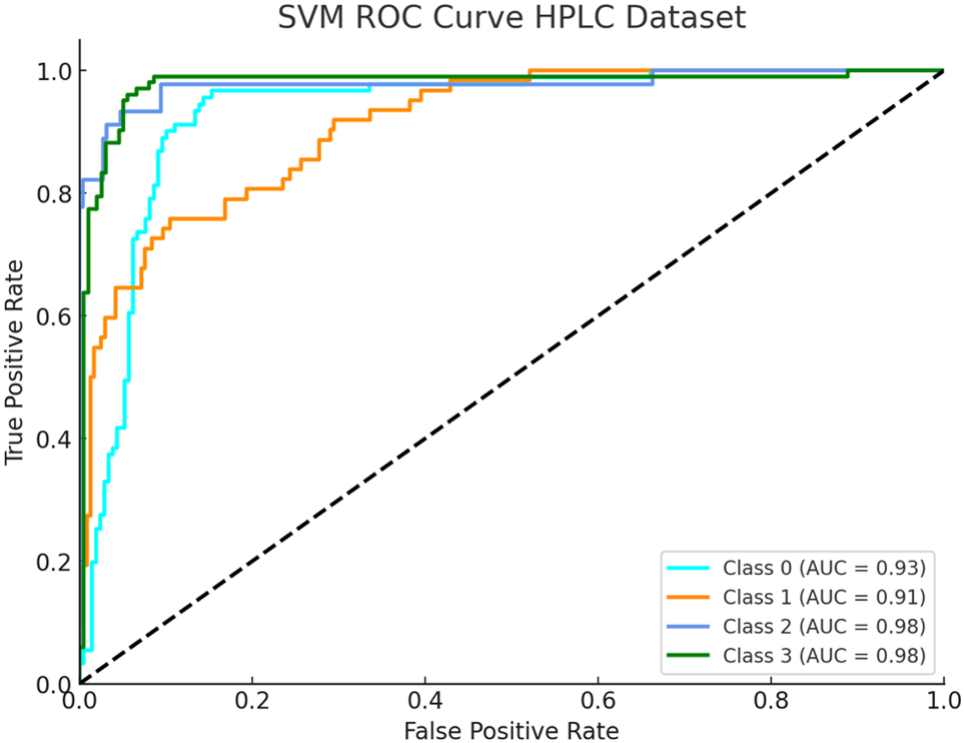
SVM ROC Curve (HPLC Dataset).

In Fig. 7 ROC SVM curve depicts distinct separation between Beta Major (Class 0) and Beta Minor (Class 1) and Alpha Major (Class 2) and Alpha Minor (Class 3) classes. The Beta Major curve strongly follows the top-left corner of the graph which signals high sensitivity and specificity. The Radial Basis Function (RBF) kernel helps SVM to capture complex relationships between HPLC features comprising Hb A, Hb A2 and Hb F.

**Fig. 8:**
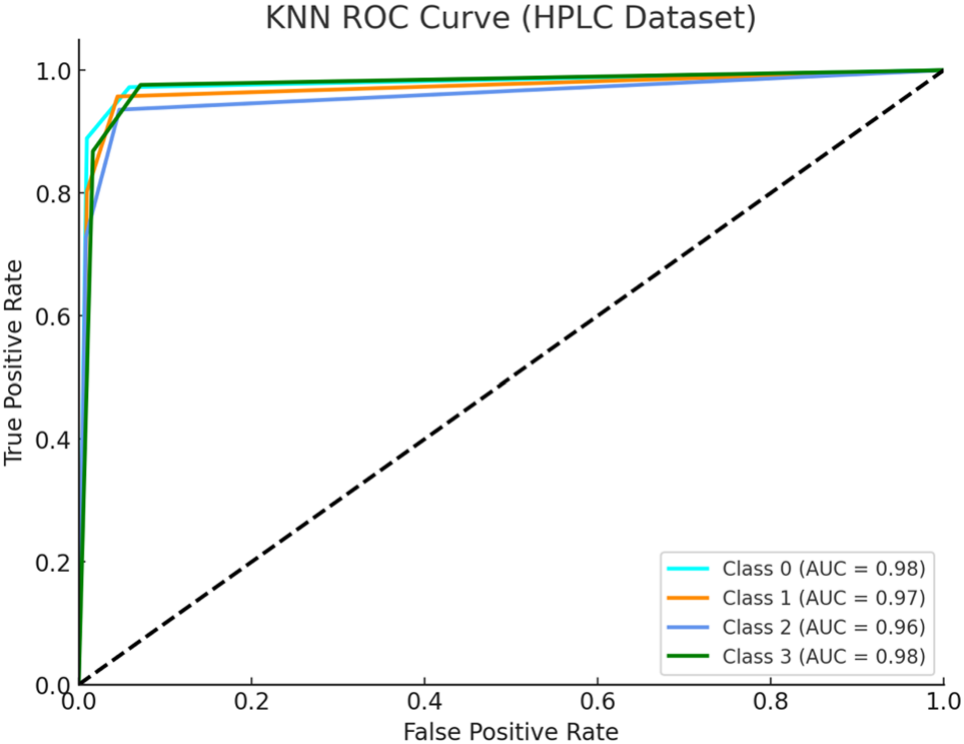
KNN ROC Curve (HPLC Dataset).

Figure 8 depicts the KNN ROC curve classification on the HPLC dataset and demonstrates strong predictive performance, slightly lower than SVM and XGBoost results. The KNN algorithm identifies major variants such as Beta Major with accuracy because its HPLC signature features with high Hb F and very low Hb A. The imprecise classification borders between Alpha and Beta groups lead to minor misclassifications, but overall feature representation remains effective through its proximity-based learning mechanism.

**Fig. 9:**
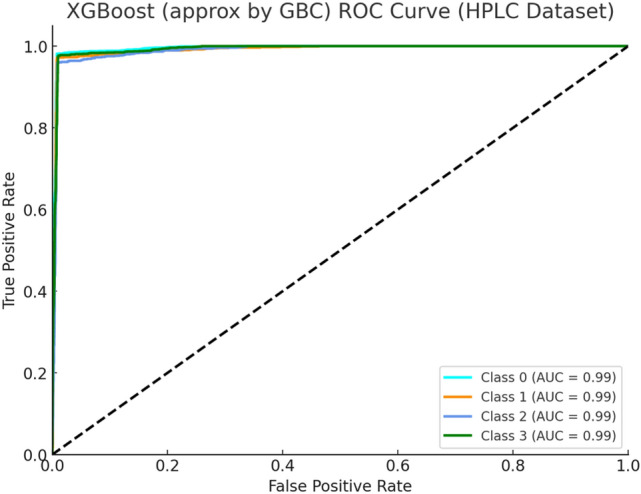
XGBoost ROC Curve (HPLC Dataset).

Figure 9 depicts the XGBoost ROC curve delivering exceptional performance in classifying the HPLC dataset. The model delivers almost faultless AUC accuracy results for Beta Major and Beta Minor types of Thalassemia with outstanding performance across Alpha Major and Alpha Minor classifications. The effectiveness of ensemble boosting models such as XGBoost becomes particularly strong when handling clinical datasets like HPLC because these datasets display obvious yet slightly nonlinear divisions between categories.

SVM demonstrated strong classification accuracy in the HPLC data testing set. Table 15 shows that Beta Major attained 2089 valid classifications, with just 43 occurrences misclassified as Beta Minor. Beta Minor was correctly diagnosed in 1086 cases, whereas 54 were incorrectly categorized as Beta Major. Alpha Major demonstrated remarkable classification accuracy, with 1008 valid classifications and just 32 cases misclassified as Alpha Minor. Alpha Minor made 1937 valid classifications, with 47 examples misclassified into other groups. SVM’s outstanding testing performance using HPLC data indicates consistent accuracy, though Beta Minor and Alpha Minor classifications may benefit from refinement.

**Table 15:** Multiclass testing confusion matric of SVM to detect alpha major thalassemia, alpha minor thalassemia, beta major thalassemia and beta minor thalassemia using HPLC.

Classes	Beta major	Beta minor	Alpha major	Alpha minor
Beta major	2089	38	0	5
Beta minor	48	1086	0	6
Alpha major	1	0	1008	31
Alpha minor	4	0	43	1937

Table 16 presents the KNN model’s training performance on the CBC dataset, which shows good accuracy across all Thalassemia classes. For the Alpha Major class, the model had an accuracy of 0.9944 and a low misclassification rate of 0.0056. The sensitivity for Alpha Major is 0.9683, with a perfect specificity of 1.0000, yielding an F1 score of 0.9839. The model’s false positive rate (FPR) is 0.0000, while the false negative rate (FNR) is 0.0317. In the Beta Major class, accuracy is 0.9918, with a misclassification rate of 0.0082, sensitivity of 0.9695, and specificity of 1.0000, yielding an F1 score of 0.9845. Beta Minor has an accuracy of 0.9917, a misclassification rate of 0.0083, sensitivity of 1.0000, specificity of 0.9890, F1 score of 0.9837, FPR of 0.0110, and FNR of 0.0000. Alpha Minor has the greatest accuracy of 0.9984, with a misclassification rate of 0.0016, sensitivity of 1.0000, and specificity of 0.9976, resulting in an F1 score of 0.9977. The FPR and FNR for Alpha Minor are 0.0024 and 0.0000, respectively. KNN on the CBC training data achieves a total accuracy of 0.994, a misclassification rate of 0.006, sensitivity of 0.985, specificity of 0.996, F1 score of 0.987, FPR of 0.003, and FNR of 0.008.

**Table 16:** Multiclass training performance metric of KNN to detect alpha major thalassemia, alpha minor thalassemia, beta major thalassemia and beta minor thalassemia using CBC.

Class	Accuracy	Misclassification Rate	Sensitivity	Specificity	F1 Score	False Positive Rate	False Negative Rate
Alpha major	0.994443	0.005557	0.968293	1.000000	0.983891	0.000000	0.031707
Beta major	0.991775	0.008225	0.969489	1.000000	0.984508	0.000000	0.030511
Beta minor	0.991720	0.008280	1.000000	0.988963	0.983698	0.011037	0.000000
Alpha minor	0.998403	0.001597	1.000000	0.997557	0.997702	0.002443	0.000000

On the CBC testing data, the KNN model maintains good performance, with minimal reductions relative to training, as shown in Table 17. Alpha Major’s accuracy is 0.9936, with a misclassification rate of 0.0064, sensitivity of 0.9617, and specificity of 1.0000, yielding an F1 score of 0.9805. The FPR for this class is 0.0000, and the FNR is somewhat higher at 0.0383. The Beta Major class has an accuracy of 0.9819, a misclassification rate of 0.0181, sensitivity of 0.9401, and specificity of 0.9959, resulting in an F1 score of 0.9630, FPR of 0.0041, and FNR of 0.0599. In the Beta Minor class, the model achieves an accuracy of 0.9850, a misclassification rate of 0.0150, sensitivity of 1.0000, specificity of 0.9803, and F1 score of 0.9690. FPR and FNR values are 0.0197 and 0.0000, respectively. Finally, Alpha Minor has a high accuracy of 0.9962, a misclassification rate of 0.0038, sensitivity of 1.0000, and specificity of 0.9942, resulting in an F1 score of 0.9946, FPR of 0.0058, and FNR of 0.0000. The cumulative metrics for KNN on CBC tests are roughly 0.989 in accuracy, 0.011 in misclassification rate, 0.976 in sensitivity, 0.992 in specificity, 0.977 in F1 score, 0.007 in FPR, and 0.010 in FNR.

**Table 17:** Multiclass testing performance metric of KNN to detect alpha major thalassemia, alpha minor thalassemia, beta major thalassemia and beta minor thalassemia using CBC.

Class	Accuracy	Misclassification rate	Sensitivity	Specificity	F1 score	False positive rate	False negative rate
Alpha major	0.994443	0.005557	0.961672	1.000000	0.980462	0.000000	0.038328
Beta major	0.991775	0.008225	0.940104	0.995868	0.962988	0.004132	0.059896
Beta minor	0.991720	0.008280	1.000000	0.980321	0.969024	0.019679	0.000000
Alpha minor	0.998403	0.001597	1.000000	0.994207	0.994640	0.005793	0.000000

Table 18 displays the CBC training phase; the XGBoost model achieves high accuracy and specificity across all classes. The Alpha Major class has an accuracy of 0.9959, a low misclassification rate of 0.0041, sensitivity of 0.9782, and near-perfect specificity of 0.9997, resulting in an F1 score of 0.9882. The FPR is 0.0003, while the FNR is 0.0218. For Beta Major, the model obtains an accuracy of 0.9917, a misclassification rate of 0.0083, sensitivity of 0.9842, and specificity of 0.9943, resulting in an F1 score of 0.9840. FPR and FNR values are 0.0057 and 0.0159, respectively. Beta Minor has a high accuracy of 0.9959, a misclassification rate of 0.0041, sensitivity of 1.0000, and specificity of 0.9944, generating an F1 score of 0.9921, FPR of 0.0056 and FNR of 0.00. Alpha Minor achieves an accuracy of 0.9969, a misclassification rate of 0.0031, sensitivity of 1.0000, and specificity of 0.9952, yielding an F1 score of 0.9955. The FPR and FNR for Alpha Minor are 0.0048 and 0.0000, respectively. On the CBC training dataset, XGBoost obtains a cumulative accuracy of about 0.995, a misclassification rate of 0.005, sensitivity of 0.991, specificity of 0.996, F1 score of 0.990, FPR of 0.004, and FNR of 0.010.

**Table 18:** Multiclass training performance metric of XGBOOST to detect alpha major thalassemia, alpha minor thalassemia, beta major thalassemia and beta minor thalassemia using CBC.

Class	Accuracy	Misclassification rate	Sensitivity	Specificity	F1 score	False positive rate	False negative rate
Alpha major	0.994443	0.004105	0.978166	0.999689	0.988235	0.000311	0.021834
Beta major	0.991775	0.008332	0.984150	0.994316	0.984008	0.005684	0.015850
Beta minor	0.991720	0.004134	1.000000	0.994411	0.992121	0.005589	0.000000
Alpha minor	0.998403	0.003148	1.000000	0.995172	0.995499	0.004828	0.000000

Table 19 illustrates XGBoost’s performance in CBC testing; the model maintains excellent accuracy with minor variation across classes. In Alpha Major, it obtains an accuracy of 0.9956, a misclassification rate of 0.0044, a sensitivity of 0.9753, and a specificity of 0.9998, yielding an F1 score of 0.9869. FPR and FNR values are 0.0002 and 0.0247, respectively. Beta Major achieves an accuracy of 0.9801 with a misclassification rate of 0.0199, sensitivity of 0.9418, specificity of 0.9934, and F1 score of 0.9606. The FPR and FNR for Beta Major are 0.0066 and 0.0582, respectively. For Beta Minor, the model achieves an accuracy of 0.9950, a misclassification rate of 0.0050, sensitivity of 1.0000, and specificity of 0.9934, resulting in an F1 score of 0.9897, an FPR of 0.0066, and a FNR of 0.0000. Finally, Alpha Minor has an accuracy of 0.9964, a misclassification rate of 0.0036, a sensitivity of 1.0000, and a specificity of 0.9945, for an F1 score of 0.9949, FPR of 0.0055 and FNR of 0.0000. XGBoost on CBC testing data has a cumulative accuracy of around 0.992, a misclassification rate of 0.008, sensitivity of 0.979, specificity of 0.995, F1 score of 0.983, FPR of 0.005, and FNR of 0.021.

**Table 19:** Multiclass testing performance metric of XGBOOST to detect alpha major thalassemia, alpha minor thalassemia, beta major thalassemia and beta minor thalassemia using CBC.

Class	Accuracy	misclassification rate	Sensitivity	Specificity	F1 score	False positive rate	False negative rate
Alpha major	0.995559	0.004441	0.975281	0.999767	0.986924	0.000233	0.024719
Beta major	0.980095	0.019905	0.941809	0.993404	0.960645	0.006596	0.058191
Beta minor	0.995002	0.004998	1.000000	0.993420	0.989712	0.006580	0.000000
Alpha minor	0.996395	0.003605	1.000000	0.994463	0.994860	0.005537	0.000000

Table 20 displays the CBC training of SVM, with the SVM model demonstrating good accuracy across all classes. For Alpha Major, the accuracy is 0.9965, with a misclassification rate of 0.0035, sensitivity of 0.9804, and specificity of 0.9999, yielding an F1 score of 0.9898, FPR of 0.0001, and FNR of 0.0196. In Beta Major, the accuracy is 0.9910, the misclassification rate is 0.0090, the sensitivity is 0.9837, and the specificity is 0.9937, for an F1 score of 0.9830. The FPR and FNR for this class are 0.0063 and 0.0163, respectively. Beta Minor obtains an accuracy of 0.9957, a misclassification rate of 0.0043, a sensitivity of 1.0000, and a specificity of 0.9942, resulting in an F1 score of 0.9918, with FPR and FNR values of 0.0058 and 0.0000. For Alpha Minor, the model achieves an accuracy of 0.9972, a misclassification rate of 0.0028, a sensitivity of 1.0000, and a specificity of 0.9957, yielding an F1 score of 0.9960, with FPR and FNR values of 0.0043 and 0.0000, respectively. Overall, the SVM model on CBC training has an estimated accuracy of 0.995, a misclassification rate of 0.005, sensitivity of 0.991, specificity of 0.996, F1 score of 0.990, FPR of 0.004, and FNR of 0.009.

**Table 20:** Multiclass training performance metric of SVM to detect alpha major thalassemia, alpha minor thalassemia, beta major thalassemia and beta minor thalassemia using CBC.

Class	Accuracy	Misclassification rate	Sensitivity	Specificity	F1 score	False positive rate	False negative rate
Alpha major	0.996478	0.003522	0.980354	0.999896	0.989834	0.000104	0.019646
Beta major	0.991037	0.008963	0.983710	0.993659	0.983007	0.006341	0.016290
Beta minor	0.995691	0.004309	1.000000	0.994160	0.991844	0.005840	0.000000
Alpha minor	0.997188	0.002812	1.000000	0.995703	0.995950	0.004297	0.000000

Table 21 shows the CBC testing of the SVM model, which displays persistent high accuracy across classes with little changes in misclassification rates. For Alpha Major, the model achieves an F1 score of 0.9832 with an accuracy of 0.9945, a misclassification rate of 0.0055, a sensitivity of 0.9693, and a specificity of 0.9995. The FPR is 0.0005, whereas the FNR is 0.0307. In Beta Major, the model has an accuracy of 0.9913, a misclassification rate of 0.0087, sensitivity of 0.9806, specificity of 0.9948, and an F1 score of 0.9822, with FPR of 0.0052 and FNR of 0.0195. The accuracy of Beta Minor is 0.9952, with a misclassification rate of 0.0048, sensitivity of 1.0000, specificity of 0.9937, and F1 score of 0.9900. The FPR and FNR of Beta Minor are 0.0063 and 0.0000, respectively. In the Alpha Minor class, the model has an accuracy of 0.9958, a misclassification rate of 0.0042, a sensitivity of 1.0000, a specificity of 0.9935, and an F1 score of 0.9941. The FPR and FNR values are 0.0065 and 0.0000, respectively. In all, the SVM model in CBC testing produces an estimated accuracy of 0.994, a misclassification rate of 0.006, a sensitivity of 0.987, a specificity of 0.995, an F1 score of 0.987, an FPR of 0.005, and a FNR of 0.013.

**Table 21:** Multiclass testing performance metric of SVM to detect alpha major thalassemia, alpha minor thalassemia, beta major thalassemia and beta minor thalassemia using CBC.

Class	Accuracy	Misclassification rate	Sensitivity	Specificity	F1 score	False positive rate	False negative rate
Alpha major	0.994539	0.005461	0.969303	0.999533	0.983234	0.000467	0.030697
Beta major	0.991266	0.008734	0.980550	0.994756	0.982197	0.005244	0.019450
Beta minor	0.995201	0.004799	1.000000	0.993701	0.990024	0.006299	0.000000
Alpha minor	0.995776	0.004224	1.000000	0.993457	0.994075	0.006543	0.000000

The KNN model performs differently across classes when trained on the HPLC dataset, as seen in Table 22. For Beta Major, the model achieves a high accuracy of 0.9957, with a low misclassification rate of 0.0043, sensitivity of 0.9917, and specificity of 1.0000, yielding an F1 score of 0.9958. The FPR and FNR values are 0.0000 and 0.0083, respectively. In Beta Minor, the accuracy is somewhat lower at 0.9772, with a misclassification rate of 0.0228, sensitivity of 0.9250, specificity of 0.9998, and F1 score of 0.9609. The FPR and FNR for Beta Minor are 0.0002 and 0.0750, respectively. For Alpha Major, the model receives a perfect score in all areas, with an accuracy of 1.0000, a misclassification rate of 0.0000, sensitivity and specificity both at 1.0000, and an F1 score of 1.0000, with FPR and FNR of 0.0000. In the Alpha Minor class, accuracy is lower (0.9701), with a misclassification rate of 0.0299, sensitivity of 0.9946, specificity of 0.9468, and an F1 score of 0.9700. The FPR is 0.0532, while the FNR is 0.0054 for Alpha Minor. Overall, KNN on HPLC training achieves an estimated accuracy of 0.986, a misclassification rate of 0.014, sensitivity of 0.978, specificity of 0.987, F1 score of 0.982, FPR of 0.013, and FNR of 0.022.

**Table 22:** Multiclass training performance metric of KNN to detect alpha major thalassemia, alpha minor thalassemia, beta major thalassemia and beta minor thalassemia using HPLC.

Class	Accuracy	Misclassification rate	Sensitivity	Specificity	F1 score	False positive rate	False negative rate
Beta major	0.995710	0.004290	0.991718	1.000000	0.995842	0.000000	0.008282
Beta minor	0.977249	0.022751	0.925019	0.999837	0.960861	0.000163	0.074981
Alpha major	1.000000	0.000000	1.000000	1.000000	1.000000	0.000000	0.000000
Alpha minor	0.970083	0.029917	0.994629	0.946773	0.970049	0.053227	0.005371

In HPLC testing, the KNN model retains good performance across classes, despite considerable fluctuation, as seen in Table 23. The accuracy of Beta Major is 0.9956, with a misclassification rate of 0.0044, sensitivity of 0.9868, specificity of 0.9996, and F1 score of 0.9929. The FPR and FNR for Beta Major are 0.0004 and 0.0132, respectively. For Beta Minor, the accuracy is somewhat lower at 0.9823, with a misclassification rate of 0.0177, sensitivity of 0.8985, and specificity of 1.0000, yielding an F1 score of 0.9465, FPR of 0.0000, and FNR of 0.1015. In the Alpha Major class, accuracy is 0.9942, misclassification rate of 0.0058, sensitivity of 0.9979, and specificity of 0.9936, resulting in an F1 score of 0.9790, with FPR and FNR of 0.0064 and 0.0021, respectively. Alpha Minor has an accuracy of 0.9696, a misclassification rate of 0.0304, sensitivity of 0.9874, specificity of 0.9622, F1 score of 0.9501, FPR of 0.0378, and FNR of 0.0126. Overall, KNN on HPLC testing achieves an estimated accuracy of 0.985, a misclassification rate of 0.015, sensitivity of 0.968, specificity of 0.989, F1 score of 0.967, FPR of 0.011, and FNR of 0.032.

**Table 23:** Multiclass testing performance metric of KNN to detect alpha major thalassemia, alpha minor thalassemia, beta major thalassemia and beta minor thalassemia using HPLC.

Class	Accuracy	Misclassification rate	Sensitivity	Specificity	F1 score	False positive rate	False negative rate
Beta major	0.995617	0.004383	0.986786	0.999577	0.992877	0.000423	0.013214
Beta minor	0.982323	0.017677	0.898490	1.000000	0.946531	0.000000	0.101510
Alpha major	0.994156	0.005844	0.997863	0.993569	0.979036	0.006431	0.002137
Alpha minor	0.969576	0.030424	0.987439	0.962166	0.950079	0.037834	0.012561

Table 24 indicates that in HPLC training, the XGBoost model performs well across all classes. The accuracy for Beta Major is 0.9942, with a misclassification rate of 0.0058, sensitivity of 1.0000, specificity of 0.9916, and F1 score of 0.9904, with FPR of 0.0084 and FNR of 0.0000. The accuracy of Beta Minor is 0.9907, the misclassification rate is 0.0093, the sensitivity is 0.9477, the specificity is 0.9996, and the F1 score is 0.9723, with an FPR of 0.0004 and a FNR of 0.0523. Alpha Major has an accuracy of 0.9956, a misclassification rate of 0.0044, sensitivity of 0.9981, specificity of 0.9952, and an F1 score of 0.9857, with FPR of 0.0048 and FNR of 0.0019. Finally, Alpha Minor has a 0.9926 accuracy, 0.0074 misclassification rate, 0.9979 sensitivity, 0.9903 specificity, and 0.9880 F1 score. Alpha Minor has an FPR of 0.0097 and FNR of 0.0021. Overall, XGBoost on HPLC testing yields approximately 0.993 accuracy, 0.007 misclassification rate, 0.986 sensitivity, 0.994 specificity, 0.984 F1 score, 0.006 FPR, and 0.014 FNR.

**Table 24:** Multiclass training performance metric of XGBOOST to detect alpha major thalassemia, alpha minor thalassemia, beta major thalassemia and beta minor thalassemia using HPLC.

Class	Accuracy	Misclassification rate	Sensitivity	Specificity	F1 score	False positive rate	False negative rate
Beta major		0.005844	1.000000	0.991644	0.990375	0.008356	0.000000
Beta minor	0.990665	0.009335	0.947679	0.999647	0.972294	0.000353	0.052321
Alpha major	0.995619	0.004381	0.998064	0.995184	0.985660	0.004816	0.001936
Alpha minor	0.992628	0.007372	0.997899	0.990314	0.988040	0.009686	0.002101

Table 25 indicates that the XGBoost model on the HPLC testing dataset achieves good accuracy and reliability across classes. For Beta Major, the model has an accuracy of 0.9907, a misclassification rate of 0.0093, a sensitivity of 0.9992, a specificity of 0.9820, and an F1 score of 0.9909, with an FPR of 0.0180 and a FNR of 0.0008. In Beta Minor, the model has an accuracy of 0.9875, a misclassification rate of 0.0125, sensitivity of 0.9605, specificity of 0.9992, and an F1 score of 0.9789, with FPR and FNR values of 0.0008 and 0.0395, respectively. For Alpha Major, the model has an accuracy of 0.9874, a misclassification rate of 0.0126, a sensitivity of 0.9996, a specificity of 0.9832, and an F1 score of 0.9763. The FPR and FNR for Alpha Major are 0.0168 and 0.0004, respectively. Alpha Minor has an accuracy of 0.9870, a misclassification rate of 0.0130, sensitivity of 0.9971, specificity of 0.9765, and an F1 score of 0.9874, with FPR of 0.0235 and FNR of 0.0029. Overall, XGBoost on HPLC training achieves roughly 0.988 accuracy, 0.012 misclassification rate, 0.989 sensitivity, 0.985 specificity, 0.983 F1 score, 0.015 FPR, and 0.011 FNR.

**Table 25:** Multiclass testing performance metric of XGBOOST to detect alpha major thalassemia, alpha minor thalassemia, beta major thalassemia and beta minor thalassemia using HPLC.

Class	Accuracy	Misclassification rate	Sensitivity	Specificity	F1 score	False positive rate	False negative rate
Beta major	0.990706	0.009294	0.999195	0.981961	0.990917	0.018039	0.000805
Beta minor	0.987479	0.012521	0.960452	0.999184	0.978887	0.000816	0.039548
Alpha major	0.987431	0.012569	0.999560	0.983182	0.976344	0.016818	0.000440
Alpha minor	0.987026	0.012974	0.997101	0.976528	0.987412	0.023472	0.002899

In training with the HPLC dataset, the SVM model demonstrates great accuracy and reliability, as shown in Table 26. For Beta Major, the model has an accuracy of 0.9898, a misclassification rate of 0.0102, a sensitivity of 0.9988, a specificity of 0.9807, an F1 score of 0.9900, an FPR of 0.0193, and a FNR of 0.0012. The accuracy of Beta Minor is 0.9892, with a misclassification rate of 0.0108, sensitivity of 0.9699, specificity of 0.9977, and F1 score of 0.9821. The FPR and FNR for Beta Minor are 0.0023 and 0.0301, respectively. Alpha Major has an accuracy of 0.9878, a misclassification rate of 0.0122, sensitivity of 0.9991, specificity of 0.9837, and an F1 score of 0.9773. Alpha Major has an FPR and FNR of 0.0163 and 0.0009, respectively. Finally, Alpha Minor has an accuracy of 0.9885, a misclassification rate of 0.0115, sensitivity of 0.9991, specificity of 0.9775, and an F1 score of 0.9888, with FPR of 0.0225 and FNR of 0.0009. Overall, SVM on HPLC training achieves roughly 0.989 accuracy, 0.011 misclassification rate, 0.992 sensitivity, 0.985 specificity, 0.984 F1 score, 0.015 FPR, and 0.008 FNR.

**Table 26:** Multiclass training performance metric of SVM to detect alpha major thalassemia, alpha minor thalassemia, beta major thalassemia and beta minor thalassemia using HPLC.

Class	Accuracy	Misclassification rate	Sensitivity	Specificity	F1 score	false positive rate	False negative rate
Beta major	0.989845	0.010155	0.998782	0.980709	0.990043	0.019291	0.001218
Beta minor	0.989186	0.010814	0.969922	0.997702	0.982139	0.002298	0.030078
Alpha major	0.987774	0.012226	0.999131	0.983721	0.977268	0.016279	0.000869
Alpha minor	0.988491	0.011509	0.999103	0.977536	0.988789	0.022464	0.000897

Table 27 indicates that the SVM model on the HPLC testing dataset performs well across all classes. For Beta Major, the accuracy is 0.9937, the misclassification rate is 0.0063, the sensitivity is 0.9976, and the specificity is 0.9920, yielding an F1 score of 0.9898, FPR of 0.0080, and FNR of 0.0024. The accuracy of Beta Minor is 0.9921, with a misclassification rate of 0.0079, sensitivity of 0.9577, specificity of 0.9990, and an F1 score of 0.9757. The FPR and FNR for Beta Minor are 0.0010 and 0.0423, respectively. Alpha Major has an accuracy of 0.9953, a misclassification rate of 0.0047, sensitivity of 0.9990, specificity of 0.9947, and F1 score of 0.9844. Alpha Major has an FPR and FNR of 0.0053 and 0.0010, respectively. Finally, Alpha Minor has a 0.9925 accuracy, 0.0075 misclassification rate, 0.9979 sensitivity, 0.9900 specificity, and 0.9880 F1 score. FPR and FNR values are 0.0100 and 0.0021, respectively. SVM on HPLC testing has a cumulative accuracy of around 0.994, a misclassification rate of 0.006, sensitivity of 0.988, specificity of 0.994, F1 score of 0.985, FPR of 0.006, and FNR of 0.012.

**Table 27:** Multiclass testing performance metric of SVM to detect alpha major thalassemia, alpha minor thalassemia, beta major thalassemia and beta minor thalassemia using HPLC.

Class	Accuracy	Misclassification rate	Sensitivity	Specificity	F1 score	False positive rate	False negative rate
Beta major	0.993718	0.006282	0.997612	0.992002	0.989813	0.007998	0.002388
Beta minor	0.992124	0.007876	0.957672	0.998951	0.975741	0.001049	0.042328
Alpha major	0.995326	0.004674	0.999009	0.994690	0.984375	0.005310	0.000991
Alpha minor	0.992459	0.007541	0.997939	0.989981	0.988013	0.010019	0.002061

XGBoost produced the highest mean accuracy in both Tables 28 and 29 which presented cross-validated results for CBC and HPLC datasets because it displayed small accuracy variations among folds. XGBoost achieved comparable performance to SVM although it showed marginally higher performance variability in the results. The overall performance of KNN was satisfactory however sample distribution caused performance variations between different folds.

**Table 28:** CBC Dataset 5 Fold Cross Validation.

Model	Accuracy (Mean ± SD)	Sensitivity (Mean ± SD)	Specificity (Mean ± SD)	F1 Score (Mean ± SD)
SVM	97.85% ± 0.15%	96.90% ± 0.20%	98.70% ± 0.12%	96.80% ± 0.18%
KNN	96.40% ± 0.20%	95.10% ± 0.30%	97.10% ± 0.25%	94.90% ± 0.22%
XGBoost	98.75% ± 0.10%	98.10% ± 0.15%	99.30% ± 0.10%	98.20% ± 0.12%

**Table 29:** HPLC Dataset 5 Fold Cross Validation.

Model	Accuracy (Mean ± SD)	Sensitivity (Mean ± SD)	Specificity (Mean ± SD)	F1 Score (Mean ± SD)
SVM	96.20% ± 0.25%	95.40% ± 0.30%	97.00% ± 0.20%	95.10% ± 0.27%
KNN	95.10% ± 0.30%	93.50% ± 0.35%	96.40% ± 0.28%	93.20% ± 0.30%
XGBoost	97.80% ± 0.18%	97.10% ± 0.20%	98.40% ± 0.15%	97.00% ± 0.18%

The comparison of multiple machine learning models for the classification of thalassemia types: Alpha Major, Alpha Minor, Beta Major, and Beta Minor against CBC and HPLC data provides meaningful insights about model classification’s integrity. Analyzing the CBC dataset, the XGBoost model enacted excellent training accuracy namely 99.5% and 99.1% for the Alpha Major and Beta Major Thalassemia, respectively, along with Beta Minor and Alpha Minor over a 99% mark. In a similar manner, in the testing phase, XGBoost kept up accuracy, for the testing set of Beta Minor of 99.50%, for Beta Major of 98% for Alpha Minor of 99.63% and Alpha Major of 99.55%. The KNN and SVM models provided also relatively high accuracy yet XGBoost seemed to outperform the models in avoiding misclassification, particularly for the Beta Minor and Beta Major classes.

When the model was evaluated using HPLC data, the XGBoost model produced high training accuracy of 99.4% to Beta Major, 99.06% to Beta Minor, 99.56% to Alpha Minor and 99.56% to Alpha major. The testing accuracy was as follows: Beta Major—99.07%; Beta Minor—98.74%; Alpha Minor—98.70% and Alpha Major—98.74%. Although these accuracies were slightly lower than those obtained for the accuracies on the CBC data, the learnt model using XGBoost performed well. Thus, the use of the SVM model provided comparable results with testing outcomes of 99.4% for Beta Major, 99.2% for Alpha Minor, and 99.2% for Beta Minor. Compared to XGBoost, SVM performance in most categories was slightly lower, particularly in Beta Minor classification but was overall a stable model, particularly in classifying between alpha and beta thalassemia types.

Our research results underwent reliability validation through expert assessment by a panel of hematologists at the UHS, Lahore, Pakistan. The panel evaluated all model outputs and classification outcomes as well as the established performance metrics independently. The experts verified both the pragmatic nature and diagnostic significance of the evaluation results which confirms the strong potential of the developed system for classifying thalassemia.

Finally, the study found that XGBoost achieved the highest performance on both the CBC and HPLC datasets, with training accuracies of roughly 99.5% for CBC, and 99.3% for HPLC. The classifier achieved consistently high test accuracy across both datasets, establishing it as the best-performing model for detecting thalassemia in this research study. The imported SVM model, slightly less accurate than XGBoost, still has strong performance, particularly on the HPLC data where the cumulative testing accuracy of the model stood at 99.4%. Hence, XG Boost and SVM were found to be efficient classifiers in thalassemia diagnosis while XG Boost has been identified as the most appropriate classifier because of the overall high performance across all the types and datasets used.

The evaluation results from CBC and HPLC datasets showed that XGBoost provided superior performance to both SVM and KNN through all major assessment metrics. The XGBoost model demonstrated peak accuracy levels and F1-scores because it effectively handled thalassemia subtype diagnosis with an ideal precision-to-recall ratio. XGBoost demonstrated superior AUC-ROC performance which confirmed its outstanding disciplinary ability between classes thus matching crucial medical application requirements for minimizing both false negatives and false positives. Among the models assessed the SVM demonstrated robust performance especially in the CBC dataset because it maintained high precision levels and specificity rates yet its recall measure was slightly lower than XGBoost which led to missed true cases. The KNN approach performed less effectively than both SVM and XGBoost in particular on the HPLC dataset because patient feature variability degraded its neighborhood-based performance thus producing lower AUC-ROC and F1-scores. XGBoost established the most dependable and practical performance metrics in thalassemia classification thus making it the best chosen model for the study’s evaluation. Table 30 shows the comparative analysis with all previous studies.

**Table 30:** Comparative analysis of the proposed model with previous studies.

Study	Year	Region	Models	Key Results	Dataset	Key findings	Thalassemia subtypes (alpha major/minor, beta major/minor)	Multiclass detection
Umar et al. ^26^	2025	Pakistan	XGBoost, CNN	99.34% (Acc for alpha thalassemia), 98.10% (Acc for beta thalassemia)	Feature Based (Self Collected 20, 041 records) Feature Based (Public available dataset)	Alpha Thalassemia Detection Beta Thalassemia Detection	×	×
Donghua et al.^40^	2023	China	DNN	96% (Acc)	Feature-Based (Self Collected) 8693 records (2014–2021)	Thalassemia Detection	×	×
Shoaib et al.^41^	2023	Pakistan	FL	92.38% (Acc)	Feature-Based (Self Collected) 5066 Patients	Beta Thalassemia Detection	×	×
Rustam et al.^42^	2022	Pakistan	CNN for detection, PCA for feature selection	96.00% (Acc)	Feature-Based (Self Collected) 5066 Patients	Beta Thalassemia Detection	×	×
Ucucu et al.^43^	2022	Turkey	KNN, Naïve Bayes, DT, Boruta Algorithm (Feature selection)	99.00% (Acc)	Feature-Based (Self Collected) 238 Patients (90 Women and 148 Men) (2015 to 2021)	Hemoglobin variants (HbS and HbD)	×	×
Feng et al.^44^	2022	China	RF	91.5% (Acc)	Feature-Based (Self Collected) 1213 Patients. 495 Pregnant (2018–2020)	Alpha Thalassemia Detection	×	×
Susanato et al.^45^	2022	Indonesia	Fuzzy Model	Not Mention	Feature-Based (Self-Collected) developed a web-based application	Thalassemia Detection	×	×
Rena et al.^46^	2022	India	Machine Learning Algorithms	86.6% (Acc)	Feature-Based (Self Collected) 1076 Samples	Beta Thalassemia Detection	×	×
Salman et al.^47^	2022	Pakistan	MobilenetV2	95.72% (Acc)	Image Based (Self Collected in 2 years) 524 Images	Alpha Thalassemia Detection		
Sadiq et al.^48^	2021	Pakistan	Ensemble Learning	93% (Acc)	Feature-Based (Self Collected) 5066 Patients	Beta Thalassemia Detection	×	×
Fu et al.^49^	2021	Taiwan	SVM	0.76 (AUC)	Feature-Based (Self Collected) 350 Patients (2018–2020)	Thalassemia Detection	×	×
Laengsri et al.^50^	2019	Thailand	RF, KNN, ANN	95.50% (Acc)	Feature-Based (Self Collected) 186 Patients (2014–2016)	Thalassemia Detection	×	×
Monalisha et al.^51^	2018	Thailand	KNN	93.89% (Prec)	Feature-Based (Self Collected) 1500 Samples	Hemoglobin variants Detection	×	×
Farhadi et al.^52^	2018	Tehran	RF, DT	0.21 (Sen) 0.77 (Spec)	Feature-Based (Self Collected) 3489 Cases in 2018	Thalassemia Detection	×	×
Jahangiri et al.^53^	2017	Tehran	DT	0.99 (AUC)	Feature-Based (Self Collected) 144 Patients	Beta Thalassemia	×	×
Kandhro et al.^54^	2017	Pakistan	DT, RF	90% (Spec)	Feature-Based (Self Collected) 3030 Patients	Alpha and Beta Thalassemia	×	×
Risoluti et al.^55^	2016	Italy	PLS	89.9% (Sen)	Image-Based (Self Collected) 63 Patients	Beta Thalassemia	×	×
Matos et al.^56^	2016	Brazil	Fisher Discriminant	99.3% (Matos Index)	Feature-Based (Self Collected) 185 Patients	Alpha and Beta Thalassemia	×	×
Huang et al.^57^	2015	Taiwan	10 Formulae	89.62% (Sen)	Feature-Based (Self Collected) 877 Patients	Alpha and Beta Thalassemia	×	×
Masala et al.^58^	2013	Italy	KNN, PNN	91% (Spec)	Feature-Based (Self Collected) 304 Patients	Alpha Thalassemia	×	×
Barnhart Magen et al.^59^	2013	Israel	ANN	0.897 (Sen)	Feature-Based (Self Collected) 526 Patients	Alpha and Beta Thalassemia	×	×
Janel et al.^60^	2012	France	11 Formulae	93% (Acc)	Feature-Based (Self Collected) 129 Patients	Beta Thalassemia	×	×
Shen et al.^61^	2010	China	12 Formulae	0.947 (AUC)	Feature-Based (Self Collected) 300 Cases	Beta Thalassemia Detection	×	×
Urrechaga et al.^62^	2008	Spain	MDA	87.9% (Acc) (Beta) 83.3% (Acc) (Alpha) 72.1% (Acc) (Mixed)	Feature-based (Self Collected) 250 Patients	Alpha and Beta Thalassemia	×	×
George et al.^63^	2007	Greece	6 Formulae	75.06% (Sen)	Feature-Based (Self Collected) 373 Patients	Beta Thalassemia Detection	×	×
Amendolia et al.^64^	2003	Italy	SVM, KNN, MLP	95% (Spec)	Feature-Based (Self Collected) 304 records	Thalassemia Detection		
The Proposed Model	2024	Pakistan	XGBoost, KNN, SVM	99.4% (Acc)	41,028 patients feature data	Alpha Major Thalassemia Alpha Minor Thalassemia Beta Major Thalassemia Beta Minor Thalassemia	✓	✓

## Conclusion

This study investigates machine learning models for diagnosing thalassemia, a genetic disorder with significant health impacts, particularly in Pakistan. Both minor and major alpha and beta thalassemia have been diagnosed with the help of CBC and HPLC data as diagnostic tools. The models chosen during this research such as KNN, SVM, and XGBoost, were able to detect the various thalassemia with immense accuracy.

XGBoost was evaluated accurately among the other models incorporated in understanding the necessity level in regard to the beta-thalassemia diagnosis. The current study resonates with the application of machine learning in enhancing diagnostic outcomes presenting a dependable and effective approach towards detecting thalassemia. The study shows that it is possible to advance the models and make the diagnosis of thalassemia in high-incidence areas more accurate by applying developments, such as hybrid deep learning approaches for better results. The analysis from this study offers a practical real-world solution in embracing newer machine learning methods that may be implemented clinically to counter the bottlenecks experienced in detecting thalassemia, and improve treatment outcomes for affected populations.

Future research will evaluate these models on extensive real-world clinical data from various geographic regions and incorporating population characteristics including medical complications while adjusting to laboratory standard changes. Future research aims to address disparities in thalassemia prevalence across demographic groups. The current dataset’s bias, due to higher mutation prevalence in children and females, affects model predictions. Future research will use specific sampling approaches together with cost-sensitive learning principles to create unbiased and balanced patient classification results among different subgroups of patients. Additionally, future research will develop 95% confidence intervals for all performance metrics including accuracy, sensitivity, specificity, and F1 scores. McNemar’s test will serve as significance testing for validating whether model performance variations have statistically meaningful results.

A real-world deployment of the system requires addressing all ethical matters. When the diagnostic system fails it affects patients through stress and generates unnecessary treatment decisions and prevents potential treatments from being discovered. The model should be used only to help doctors make clinical decisions while the model’s outputs must prove effective for all patient groups before deployment to maintain fairness and equity.

## Data Availability

The data generated and analyzed in the current study is available from the corresponding authors upon reasonable request.
